# Activable Photodynamic DNA Probe with an “AND” Logic Gate for Precision Skin Cancer Therapy

**DOI:** 10.34133/research.0295

**Published:** 2024-01-24

**Authors:** Jiaojiao Zhu, Lanyuan Peng, Shah Jehan, Haiyang Wang, Xiang Chen, Shuang Zhao, Wenhu Zhou

**Affiliations:** ^1^ Xiangya School of Pharmaceutical Sciences, Central South University, Changsha, Hunan 410013, China.; ^2^ Department of Dermatology, Hunan Engineering Research Center of Skin Health and Disease, Hunan Key Laboratory of Skin Cancer and Psoriasis, Xiangya Hospital, Central South University, Changsha, Hunan 410008, China.; ^3^ Key Laboratory of Biological Nanotechnology of National Health Commission, Changsha, Hunan 410008, China.; ^4^ Furong Laboratory, Changsha, Hunan, China.; ^5^ Department of Vascular Surgery, The First Affiliated Hospital of Guangzhou Medical University, Guangzhou, Guangdong 510120, China.

## Abstract

Photodynamic therapy (PDT) has emerged as a promising approach for squamous cell carcinoma treatment but hindered by tumor hypoxia, acquired resistance, phototoxicity, and so on. To address these issues, we developed a smart strategy utilizing activable photosensitizers delivered by an aptamer-functionalized DNA probe (ADP). The ADP incorporated an AS1411 aptamer for tumor targeting and a linear antisense oligonucleotide (ASO) for recognition of Survivin mRNA. In the absence of the target, PDT remained quenched, thereby avoiding phototoxicity during circulation and nonselective distribution. With the aid of the aptamer, ADP achieved selective targeting of tumors. Upon internalization, ADP targeted recognized Survivin mRNA, triggering PDT activation, and releasing ASO to down-regulate Survivin expression and reverse tumor resistance. Consequently, the activable photosensitizers exhibited an “AND” logic gate, combining tumor-targeting delivery and tumor-related gene activation, thus enhancing its specificity. Additionally, the incorporation of hemin into the ADP provided catalase activity, converting tumor-abundant H_2_O_2_ into O_2_, thereby ameliorating tumor hypoxia. The resulting functionalized G-quadruplex/hemin–DNA probe complex demonstrated targeted delivery and activation, minimized side effects, and enhanced PDT efficacy in both xenograft tumor-bearing mice and patient-derived xenograft models. This study offers a unique and promising platform for efficient and safe PDT, thus holding great potential for future clinical translation and improved cancer therapy.

## Introduction

Cutaneous squamous cell carcinoma (SCC) is the second most prevalent skin cancer, and its incidence has shown a consistent increase over time [1–3]. Despite the availability of conventional therapies such as surgery, chemotherapy, and radiotherapy, the outcomes for SCC treatment often remain unsatisfactory, particularly in cases where treatment challenges arise due to patient factors or the tumor’s specific location, necessitating tissue preservation. Therefore, there is an urgent need for nonsurgical therapeutic approaches to improve SCC treatment outcomes [3–5]. In recent years, photodynamic therapy (PDT) has emerged as a promising modality for various cancers, including SCC, due to its advantages of superficial tissue penetration, precise spatiotemporal control, and minimal side effects, making it particularly suitable for skin cancer treatment. PDT has shown promise not only in treating SCC but also in preventing its occurrence, such as in the treatment of actinic keratosis, an early manifestation of SCC [4]. However, achieving optimal efficacy with PDT poses challenges, including tumor hypoxia, acquired resistance, and phototoxicity to nearby normal tissues [6,7].

To enhance the selectivity and efficacy of PDT, researchers have explored smart drug delivery systems that enable targeted delivery and controlled activation of photosensitizers (PS) [8–11]. Two critical aspects of effective SCC therapy are the selective accumulation of PS in tumor cells and maintenance of an adequate oxygen supply within the tumor microenvironment [4]. Nonselective distribution of PS to both tumor and normal cells can reduce therapeutic efficacy and lead to unintended side effects [12]. Additionally, tumor tissues often exhibit hypoxia, and PDT further depletes oxygen, compromising its effectiveness [13–15]. Furthermore, PDT may induce overexpression of resistance genes, such as Survivin, which can hinder the apoptotic effects and regulate cell division, contributing to PDT resistance [16]. To address these challenges, researchers have designed intelligent drug delivery systems to enhance PS accumulation and reverse tumor resistance, thereby improving PDT efficacy [6,14,17,18]. Nevertheless, these approaches have primarily focused on improving PDT efficacy, with limited attention given to mitigating phototoxicity.

Recent advancements in activable PS have offered a promising strategy for targeted and effective cancer therapy with decreased phototoxicity [10,19–22]. Activable PS remains inactive during systemic circulation and in normal tissues but becomes activated specifically within tumor cells in response to tumor-associated stimuli, such as specific mRNA. This selective activation allows for precise and targeted PDT for cancer treatment [23,24]. The concept of activable PS is reminiscent of the DNA probe (DP) for labeling and detecting specific target molecules. Nucleic acid probes, in particular, have gained widespread use in biomedical research due to their high specificity, sensitivity, biocompatibility, and ease of synthesis. Its versatility lies in its integration with various functional nucleic acids, such as aptamers and DNAzymes, enabling theranostic applications [19,25–28]. In our previous study, an aptamer-functionalized DNA probe (ADP) was introduced, capable of delivering DP to tumor cells and responding to Survivin mRNA, leading to tumor imaging and selective inhibition of tumor growth [29]. This underscored the potential of functional DP as a platform for tumor-selective PS delivery with enhanced therapeutic efficacy [9,25].

**Fig. 1.**
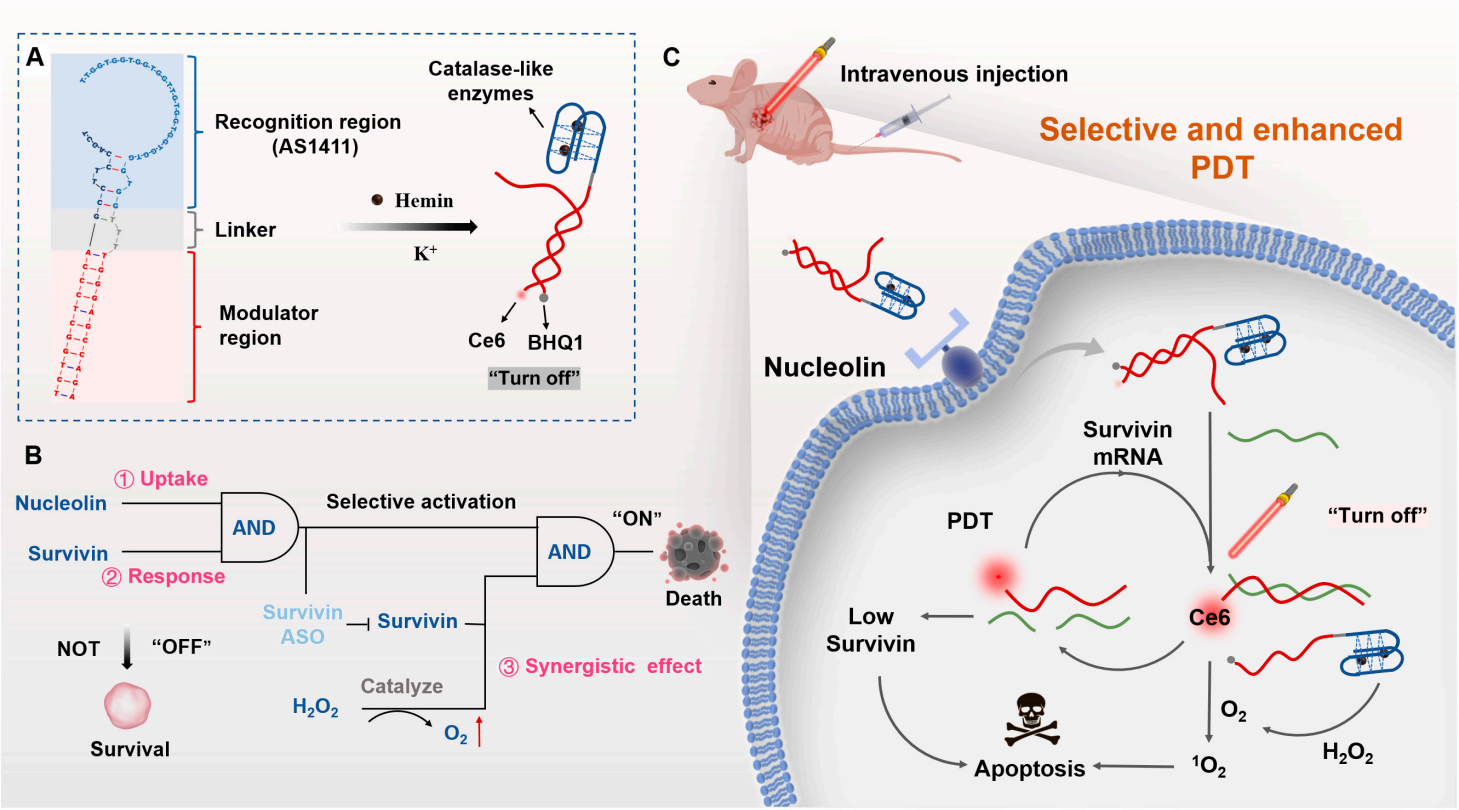
Project design diagram. (A) The DNA sequence and secondary structure of the G4DP. (B) The schematic illustration of “AND” logic gate of G4DP in response to nucleolin and Survivin for precision tumor therapy. (C) The in vivo application of G4DP for targeted skin cancer therapy with enhanced PDT efficacy via Survivin down-regulation and self-oxygen supply.

Inspired by this concept, we have employed the concept of ADP to deliver PS for PDT of SCC. The designed structure of ADP includes an AS1411 aptamer, which targets nucleolin expressed on various tumor cells [8,9,30], and a linear DP specific to Survivin mRNA (Fig. 1A). This DP consists of 2 single-stranded DNA with attached PS Ce6 and a quencher molecule at each end. In the absence of the target (Survivin mRNA), the probe is in close proximity, resulting in quenching of PDT. Upon targeted delivery into tumor cells mediated by the AS1411 aptamer, the DP hybridizes with high levels of Survivin mRNA, leading to probe separation, Ce6 release from the quencher, fluorescence signal, and PDT activation (Fig. 1B). Additionally, the hybridization of the probe DNA with Survivin mRNA acts as an antisense oligonucleotide (ASO) to down-regulate Survivin expression, thus reversing tumor resistance to PDT (Fig. 1C). Furthermore, hemin is incorporated into the AS1411 structure to form a G-quadruplex/hemin complex, exhibiting catalase activity to convert tumor-abundant H_2_O_2_ into O_2_, addressing the limitation of PDT caused by tumor hypoxia [17,25,31].

The resulting G-quadruplex/hemin-DP complex, termed G4DP, demonstrates capabilities for tumor-targeted delivery, activation with turn-on signal, and PDT enhancement through Survivin down-regulation and hypoxia relief. We comprehensively investigated the in vitro and in vivo performances of G4DP, demonstrating its enhanced therapeutic effect, minimal side effects, and biosafety in both xenograft tumor-bearing mice and patient-derived xenograft (PDX) models. These results highlight the superiority of G4DP over conventional PDT strategies, making it a promising and efficient therapeutic agent for SCC treatment with the potential for future clinical translation.

## Materials and Methods

### Materials and reagents

All oligonucleotides were purchased from Tsingke Biotechnology Co., Ltd. (Beijing, China), Ce6-labeled oligonucleotides were purchased from Takara Bio Inc. (Beijing, China), and the sequences are listed in Table S1. Hemin was purchased from Sigma-Aldrich Co., Ltd (St. Louis, MO, USA). Potassium chloride (KCl) and sodium chloride (NaCl) were purchased from Sinopharm Chemical Reagent Co., Ltd. (Shanghai, China). Hemin, 3,3′,5,5′-tetramethyl-[1,1′-biphenyl]-4,4′-diamine, 2,7-dichlorodi-hydrofluorescein diacetate (DCFH-DA), and singlet oxygen sensor green (SOSG) probe were obtained from Macklin Co., Ltd (Shanghai, China). Hepes was purchased from BioFroxx (China). The antibodies of Nucleolin-Alexa Fluor 488 conjugate, HIF-1α (hypoxia-inducible factor-1 alpha), Survivin, SERPINB3 (serpin peptidase inhibitor, clade B [ovalbumin], member 3), β-actin, glyceraldehyde-3-phosphate dehydrogenase, and goat anti-rabbit IgG (immunoglobulin G)-Alexa Fluor 488 conjugate antibodies were from Bioss (Beijing, China), Cell Signaling Technology (USA), and Proteintech (Wuhan, China), respectively. All reagents used in the cellular experiment were purchased from Gibco Life Technologies (Grand Island, NY, USA). All chemical reagents were in analytical purity and were used without further purification. Solutions were prepared with diethyl pyrocarbonate water (Biosharp Life Sciences, China).

### Preparation of ADPs

In this project, a BHQ-1-labeled DNA aptamer AS1411 hybridized with Ce6-labeled ASOs of the Survivin gene. We predicted the secondary structure of ADPs by using the Mfold web server for hybridization Fig. S1 [32]. Both DNA strands BHQ-1- and Ce6-labeled (BHQ-1:Ce6 = 1.5:1) were mixed in Hepes buffer (10 mM, pH 7.4, containing 100 mM KCl and 10 mM NaCl). Consequently, the mixture was heated to 95 °C for 5 min and then annealed by cooling to 25 °C. After hybridization, the ADP was incubated with hemin (BHQ-1:hemin = 1:2) for 2 h in the dark at room temperature. Different ADPs were termed as shown in Table S2.

### ADPs screening for sensor performance and ADP structural stability in vitro

To optimize the specific response performance of ADPs to the substrate, the base complementary length between strand #1 and strand #2 was changed (*n* = 7, 9, and 11) and a FAM/BHQ-1 fluorescence pair was used (use FAM instead of Ce6). First, DNA strand #1 and strand #2 were hybridized to form an ADP correctly, and the fluorescence of the solution system was measured by a fluorescence microplate reader (Infinite M200, Tecan). Next, the target DNA was added and incubated for 30 min, and the fluorescence intensity of the solution system was measured again. To evaluate the response dynamics of ADP, the restored fluorescence intensity (Ex/Em = 470/518 nm) at different time points was measured after adding target DNA (strand #1:strand #2:target DNA = 1.5:1:2).

To evaluate the sensor capabilities of ADP-11bp, various concentrations of target DNA were incubated with 50 nM of different ADPs at 37 °C for 30 min. The fluorescence intensities were measured with wavelengths of Ex/Em = 404/663 nm. Plotting the Δpeak intensity (with/without target) at 663 nm against the target concentration generated a linear curve within the range of 0 to 300 nM target DNA and determined the limit of detection based on the 3σ/slope calculation (σ = background variation of the sensor without target DNA). To evaluate the sensitivity of ADP, 300 nM of target DNA, misDNA-1, misDNA-2, has-Mir-942, and has-Mir-1247-3p were used.

To measure the ^1^O_2_ production after laser irradiation, the SOSG probe was employed. Briefly, different samples, which contained the same concentration of Ce6 (1 or 0.5 μM), were mixed with the SOSG probe (2.5 μM), and the fluorescence intensity (Ex/Em = 490/525 nm) of each sample at different time points was detected with/without H_2_O_2_ (100 mM) and a continuous laser at 630 nm, 0.1 W/cm^2^ for 10 min.

To measure the catalase-like activity of G-quadruplex/hemin DNAzyme (G4/hemin for short), different samples (1.5 μM in G4/hemin) was dispersed in the H_2_O_2_ (100 mM) and the oxygen level of every sample at different time points was detected by the portable dissolved-oxygen meter (JPBJ-609L, INESA Scientific Instrument Co., Ltd., China).

To investigate the structural stability of DP structures in vitro, we dispersed G4DP (2 μM in Ce6) in various physiological simulation solutions, including a 10% fetal bovine serum (FBS) solution, a 0.1-U deoxyribonuclease (DNase) solution, and a mixture of the two (dispersed in pH 7.4 tris buffer containing Ca^2+^ and Mg^2+^), and incubated them at 37 °C for varying durations. Subsequently, we terminated the reaction by adding protease K, EDTA, and 0.1% sodium dodecyl sulfate, followed by further incubation at 37 °C for 30 min. The resulting degradation of DP was then analyzed using a 15% native-polyacrylamide gel electrophoresis gel.

### Cell surface expression of Nucleolin

To assess the expression of nucleolin on the cell surface, a Nucleolin-Alexa Fluor 488 conjugate antibody specific to human nucleolin was used. Briefly, different cells (including A375, A431, HaCaT, HUVEC, and L929 cells) were seeded into a 12-well plate and cultured overnight. The cells were washed 3 times with precooled phosphate-buffered saline (PBS) and fixed with paraformaldehyde (4%) for 15 min. Next, 5% bovine serum albumin (BSA) in PBS was added and incubated at room temperature for 30 min. Then, the BSA was replaced with a 1:100 dilution of Alexa Fluor 488 conjugate antinucleolin antibody in 1% BSA PBS solution and incubated at 4 °C for 1 h. Next, cells were washed twice with precooled PBS and added with 4′,6-diamidino-2-phenylindole (1 μg/ml) reagent for 15 min. Finally, the cells were immersed in the fluorescent antiquenching agent and observed by a fluorescence microscope.

### In vitro cellular uptake of ADPs

To evaluate the selectivity of ADPs to cell surface nucleolin and cellular Survivin, different cells (including A375, A431, HaCaT, HUVEC, and L929 cells) were seeded into a 12-well plate and cultured overnight. The cells were washed twice with PBS and incubated with different ADPs (500 nM in Ce6) in the dark condition for different time points. Subsequently, the cells were fixed with paraformaldehyde (4%) for 20 min. Then, the nuclei of cells were stained with 4′,6-diamidino-2-phenylindole (1 μg/ml) reagent for 15 min. Finally, the cells were washed twice with PBS and visualized by a confocal laser scanning microscope (CLSM, LSM780 NLO, Zeiss, Oberkochen, Germany) with wavelengths of Ex/Em = 404/663 nm.

### Western blotting analysis

The protein expression of Survivin and HIF-1α in ADPs-treated A431 cells was determined by Western blotting assay. The cells were seeded into a 6-well plate and cultured overnight. Then, the cells were treated with different ADPs (500 nM in Ce6) in the dark condition for 24 h. Next, the cells were irradiated with/without a continuous laser at 630 nm, 0.1 W cm^−2^ for 5 min. After another 24-h culture, the protein expression of Survivin and HIF-1α in different groups was determined by Western blotting assay.

### In vitro cytotoxicity studies

The cytotoxicity of different ADPs was evaluated by MTT (3-(4,5-dimethyl-2-thiazolyl)-2,5-diphenyl-2-H-tetrazolium bromide, thiazolyl blue tetrazolium bromide) assay and flow cytometry. The cells were treated as above mentioned. Subsequently, Annexin V fluorescein isothiocyanate/propidium iodide double-staining kit was used to assess the cell apoptosis and the MTT was performed to assess the cell viability.

### In vivo study for biodistribution of ADPs

Balb/c female nude mice (6 to 8 wk, 20 ± 2 g) were purchased from Tianqin Biotech. Co., Ltd. (Changsha, China) and were maintained in a specific-pathogen-free environment and allowed free access to water and food. All animal experiment protocols were reviewed and approved by the experimental animal ethics committee at Central South University (CSU-2023-0253) and were carried out following the requirements of the National Act on the use of experimental animals (People’s Republic of China).

The mice were subcutaneously inoculated on the dorsal left side with human squamous cancer cell line A431 (1 × 10^6^ cells into nu/nu mice, 0.2 ml per mouse), and tumors were allowed to establish over time. When tumor sizes reached around ~100 mm^3^, the mice were injected intravenously (iv, tail) with 0.5 nmol Cy5.5 of corresponding conjugate RDP, G4DP (without BHQ-1), and G4DP (*n* = 3). The images of all the mice were taken using an in vivo imaging system, IVIS Spectrum at 4, 8, 12, and 24 h postinjection. The images were obtained in white light and the fluorescence channel (Cy5.5 mode). After 24-h administering, the tumor and main organs (heart, liver, spleen, and kidney) were resected, and the images were taken as described above.

For further determining the pharmacokinetics behavior of ADPs in the mice, the healthy mice were injected intravenously (iv, tail) with 3-nmol Cy5.5 of corresponding conjugate G4DP (without BHQ-1) and G4DP (*n* = 3). Subsequently, the blood samples were collected from the tail and centrifuged at 3,000 rpm for 15 min to collect plasma at different time points. The plasma was then diluted 6 times with saline, and the fluorescence was measured by a microplate reader (Ex/Em = 650/695 nm) with a standard curve made from free Cy5.5-labeled strand #2.

### In vivo phototoxicity of ADPs

The tumor-bearing mice were randomly divided into 3 groups (*n* = 3) and intravenously injected with different conjugates (at a Ce6 equivalent dose of 15 nmol), respectively. The mice were subject to photoirradiation for 1 h using a light-emitting diode light source (630 nm, 10 mW/cm^2^) after 4 h postinjection. Then, the mice were fully exposed to the natural light environment. To evaluate the safety, the blood and main organs were collected after 24 h posttreatment. The levels of aspartate aminotransferase (AST), alanine aminotransferase (ALT), blood urea nitrogen (BUN), creatinine (Cre), IgG, and C-reactive protein (CRP) in the serum were determined. The main organs were stained with hematoxylin and eosin (H&E) for histological observation by optical microscope.

To further examine the phototoxicity on the skin, different conjugates were injected into each mouse subcutaneously (at a Ce6 equivalent dose of 10 nmol), followed by irradiation at the dosing sites for 10 min using laser irradiation (630 nm, 100 mW/cm^2^). Then, the mice were fully exposed to a natural light environment and the skin at the dosing sites was separated for H&E staining at 24 h postinjection.

### In vivo antitumor efficacy of ADPs

To evaluate the in vivo therapeutic efficacy of ADPs, the SCC tumor-bearing mice were established. When tumor sizes reached around ~50 mm^3^, the mice were randomly divided into 4 groups (*n* = 4) and injected intravenously (iv, tail) with 10 nmol of Ce6 of corresponding conjugate ADP-NC, ADP, and G4DP, every 3 d 3 times. After 24 h postinjection, the tumor was irradiated for 10 min (630 nm, 100 mW/cm^2^). Subsequently, the tumor volumes of mice were measured every 2 d until 14 d and the tumor volumes were calculated following the formula: volume = (tumor length) × (tumor wide)^2^/2. The relative tumor volume was calculated as* V/V0* (*V* and *V0* are the tumor volume detected at time t and t0, respectively). Then, the blood, main organs, and tumors were collected for safety evaluation and evaluation of the Survivin, HIF-1α, TUNEL (TdT-mediated dUTP nick end labeling), and Ki67 expression.

To further assess the antitumor efficacy of ADPs, the PDX model was established. First, primary SCC tumor tissues were obtained from patients in the Department of Dermatology at Xiangya Hospital, with informed consent from the patient. Subsequently, we established the PDX on the nude mice following published protocols, and histology analysis was determined for the tumor from F_3_ generation mice [33]. When the tumor volume reached ~50 mm^3^, the F_3_ generation mice were randomly divided into 4 groups (*n* = 4) and treated as above mentioned to evaluate the antitumor effect of ADPs.

### Establishment of primary SCC cells

The primary SCC tumor tissues were obtained from patients in the Department of Dermatology at Xiangya Hospital, with informed consent from the patient. All experiment protocols were reviewed and approved by the Clinical Medical Ethics Committee, Xiangya Hospital, Central South University (202305092). First, the adipose and necrotic tissue of the tumor was removed and then the tumor was cut into fragments (< 1 mm^3^) in sterile tubes. Then, 1.5 ml of PBS containing 0.2% collagenase IV (Sigma, USA) and 0.1% Dispase II enzymes (Sigma, USA) was added and incubated at 37 °C for 1 h. Subsequently, the cell supernatant was filtered through 100-mesh and 40-mesh cell screens in turn. The cell suspension was centrifuged at 400 g for 8 min at 4 °C and washed twice with D-Hank’s solution. Finally, the cell precipitation was collected and resuspended in 2 ml of complete Opti-MEM medium in a 6-well plate. The cells were incubated at 37 °C in 5% CO_2_ and the medium was refreshed every 2 d until the primary cells attach to the plate (around 7 d) [34]. Then, the immunofluorescence analysis on SERPINB3 (a biomarker of SCC) was determined for the attached primary cells, and an in vitro cell uptake experiment was carried out as above mentioned above.

### Statistical analysis

Statistical analyses were performed using GraphPad Prism 6.01 software. Student *t* test and one-way analysis of variance were used to assess the significance of differences among groups. The significance was defined as follows: *P < 0.05, **P < 0.01, ***P < 0.001, and ****P < 0.0001.

## Results and Discussion

### Structure optimization for selective Survivin mRNA sensing, PDT activation, and self-oxygenation

**Fig. 2.**
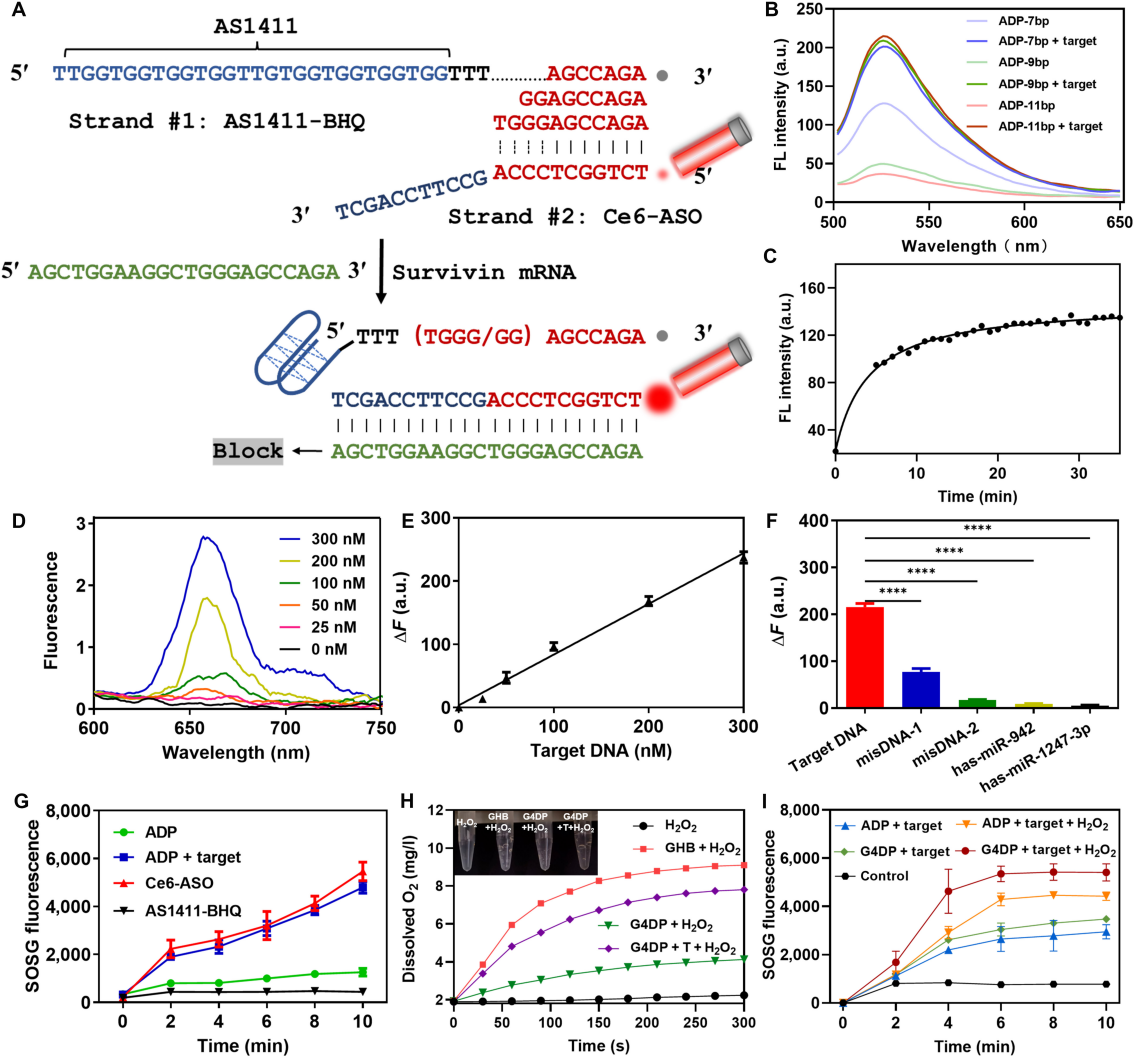
Optimization and verification of DNA probes. (A) Schematic representation of the design strategy for ADP enabling target recognition and PDT activation. (B) Responsiveness of different ADPs (*n* = 7, 9, and 11) to the target. (C) Response dynamics of ADP (*n* = 11) to the target. (D) Sensitivity of the sensor ADP (*n* = 11) to various concentrations of the target. (E) Sensor response as a function of target concentration. (F) Specificity test of the sensor using 50 nM different DNA sequences. (G) ^1^O_2_ generation from different ADPs. (H) Changes in dissolved O_2_ levels after adding different ADPs in the presence or absence of H_2_O_2_. (I) ^1^O_2_ generation from different ADPs in the presence or absence of H_2_O_2_.

The design of the ADP structure involved 2 strands: strand #1 comprised the AS1411 sequence with a 3′ terminal dark quencher BHQ-1 modification, along with an extension of several bases, while strand #2 consisted of the antisense sequence of Survivin mRNA with a 5′ terminal Ce6 modification (alternatively, FAM or Cy5.5 could replace Ce6 based on the experimental design) (Fig. 2A). Through careful sequence design, partial hybridization between these 2 strands allowed for close proximity of BHQ-1 and Ce6, resulting in fluorescence quenching in the “off” mode. Upon addition of the target, strand #2 fully complemented the target nucleic acid, leading to the dissociation of strand #2 from strand #1 and subsequent restoration of fluorescence, indicating the transition of the ADP to the “on” mode. In addition, the hybridization of strand #2 with the target mRNA served as an ASO for gene silencing.

To optimize the specific response performance of the ADP to the target, different numbers of complementary bases between strand #1 and strand #2 were tested (*n* = 7, 9, and 11). Increasing the length of complementary bases enhanced the efficiency of BHQ-1-mediated fluorescence quenching without affecting the fluorescence recovery ability of the ADP (Fig. 2B). Notably, the ADP with 11 complementary bases demonstrated the most favorable responsiveness. The response dynamics of the ADP to the target were evaluated, revealing a gradual fluorescence recovery that reached its maximum after 10 min (Fig. 2C), indicating rapid target response and ADP activation.

Subsequently, the sensitivity of the ADP was assessed by adding various concentrations of the target, resulting in a proportional increase in fluorescence (Fig. 2D). Plotting the Δpeak intensity (with/without target) at 663 nm against the target concentration generated a linear curve within the range of 0 to 300 nM target DNA (Fig. 2E). The calculated detection limit was 2.9 nM, illustrating the ADP’s excellent sensitivity to target DNA. Such sensitivity was comparable to the DP reported previously [29]. Moreover, the ADP with 11 complementary bases exhibited higher selectivity for the target, attributed to its low background signal. The specificity of the ADP was evaluated using mismatched DNAs and several microRNAs as a control. DNA with a single base mismatch (misDNA-1) produced a weak signal, while other sequences did not interfere substantially (Fig. 2F), indicating high specificity. These findings demonstrate the potential of the ADP to respond to intracellularly overexpressed Survivin gene with high sensitivity and specificity, indicating its potential for tumor diagnosis.

Additionally, we assessed the stability of ADP in a simulated physiological environment. Our results indicated that ADP maintained stability in 10% FBS for a minimum of 24 h before undergoing gradual degradation (Fig. S2). Conversely, ADP displayed relative instability in a solution containing DNase I, exhibiting noticeable degradation after a 24-h incubation period. Intriguingly, the presence of FBS notably bolstered ADP’s stability within the DNase I solution. Encouragingly, a substantial proportion of ADP remained structurally stable even after 36 h of coincubation, a critical aspect essential for subsequent in vivo applications of ADP.

To investigate the selective photodynamic effect of the ADP, Ce6-labeled ADP’s capability to generate singlet oxygen (^1^O_2_) upon near-infrared activation was studied using the SOSG probe, a crucial component for PDT. As a control, Ce6-labeled strand #2 demonstrated significant ^1^O_2_ generation within 10 min (Fig. 2G). When strand #2 was hybridized with BHQ-1-labeled strand #1 to form the ADP, the PDT effect of Ce6 was quenched by BHQ-1, indicating the “off” mode of Ce6. However, upon addition of the target, which released strand #2 and restored Ce6 fluorescence, the ^1^O_2_ generation capability of Ce6 recovered, becoming indistinguishable from free strand #2. Thus, the ADP in the “off” mode did not induce PDT effects to avoid nonspecific phototoxicity but regained its activity for tumor-specific PDT.

Given that many solid tumors exhibit hypoxia due to rapid proliferation and abnormal vasculature, oxygen becomes critical for PDT as it serves as the feedstock for PS to generate ^1^O_2_. To address this issue, various nanomaterials have been developed to alleviate the hypoxic tumor microenvironment [35,36]. One such approach involves catalase, an enzyme that can catalyze the high expression of H_2_O_2_ in the tumor site to produce O_2_, thereby mitigating tumor hypoxia and enhancing PDT efficacy [37,38]. In this study, AS1411 formed a G4 structure in the presence of K^+^ and incorporated hemin to generate a G4/hemin DNAzyme with catalase activity [9,25], resembling the function of catalase. Similar to catalase, the G4/hemin DNAzyme, as an iron-based catalyst, could facilitate the Haber–Weiss reaction, decomposing H_2_O_2_ into O_2_. This catalytic activity was expected to reinforce ADP-mediated ^1^O_2_ production and enhance therapeutic efficacy. To confirm this concept, the ability of the ADP to decompose H_2_O_2_ into O_2_ was investigated. In the presence of H_2_O_2_ solution alone (control), no O_2_ was detected (Fig. 2H). However, upon addition of K^+^/hemin to form the G4/hemin DNAzyme in strand #1 (referred to as GHB), significant bubble formation occurred, and a rapid O_2_ generation was observed, confirming the catalase-mimic activity of the G4/hemin DNAzyme. When the GHB hybridized with strand #2 to form the ADP (referred to as G4DP), the O_2_ generation was considerably decreased, likely due to the formation of the duplex strand, which reduced the flexibility of the structure and impeded catalytic activity. However, when the G4DP was combined with the target, O_2_ generation increased.

Additionally, the synergistic effect of G4/hemin on Ce6-mediated ^1^O_2_ production was investigated. In the absence of H_2_O_2_, there was minimal difference in ^1^O_2_ generation between the ADP and G4DP groups (Fig. 2I). However, upon the addition of H_2_O_2_, a significant increase in ^1^O_2_ production was observed for the G4DP, attributed to the catalase-like activity of G4/hemin. Consequently, the G4/hemin DNAzyme fragment within the G4DP structure acted as a self-oxygen generator, effectively alleviating tumor hypoxia and augmenting the therapeutic efficacy of PDT.

### Nucleolin-mediated cellular uptake and Survivin mRNA-triggered activation inside cells

**Fig. 3.**
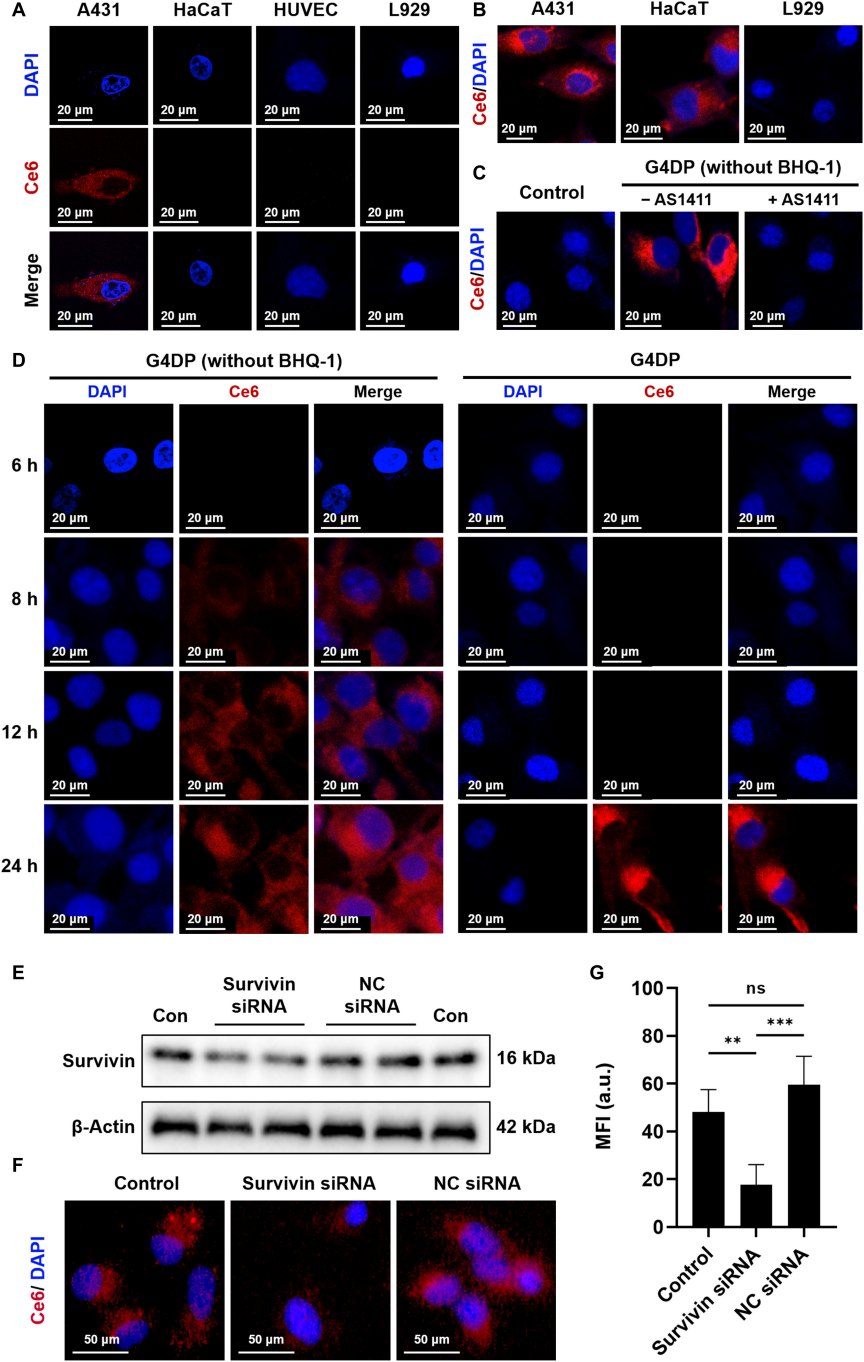
Nucleolin-mediated cellular uptake and Survivin mRNA-triggered activation inside cells. (A) Cellular uptake and activation of G4DP in different cell lines. (B) Cellular uptake of G4DP (without BHQ-1) in different cell lines. (C) Cellular uptake of G4DP (without BHQ-1) in A431 cells with or without AS1411 pretreatment. (D) Intracellular behavioral dynamics of G4DP with/without BHQ-1 in A431 cells. (E) Western blot analysis of Survivin expression in cells after different treatments. (F) Cellular uptake of G4DP in A431 cells with or without Survivin siRNA pretreatment, and (G) mean fluorescence intensity of Ce6 uptake. *P < 0.05, **P < 0.01, ***P < 0.001, and ****P < 0.0001.

To achieve selective PDT in tumor cells while minimizing toxicity to normal cells, the G4DP structure was designed with dual responsivity, utilizing AS1411-mediated cellular uptake targeting nucleolin and Survivin-triggered activation. Five different cell lines were selected based on their expression levels of nucleolin and Survivin. A431 and A375, both skin carcinoma cell lines, exhibited high levels of surface nucleolin and Survivin expression. HaCaT, an immortalized keratinocyte cell line, showed minimal surface nucleolin expression and nearly no Survivin expression, serving as a representative of normal human keratinocytes. Human umbilical vein endothelial cells (HUVEC), characterized by low surface nucleolin and Survivin expression, and L929, a nucleolin-negative and human Survivin-negative murine fibroblast cell line, were also included (Fig. S3). Confocal microscopy was employed to evaluate the responsiveness of G4DP in these cell lines. As anticipated, fluorescence signals from G4DP were only observed in A431 cells (Fig. 3A), indicating successful activation, while cell lines lacking sufficient surface nucleolin or Survivin expression (HaCaT, HUVEC, and L929) did not exhibit G4DP activation.

To confirm the AS1411-mediated cellular uptake, we modified G4DP by excluding BHQ-1 to allow continuous fluorescence of Ce6, enabling tracking of G4DP delivery. Remarkable Ce6 fluorescence was observed in nucleolin-positive cell lines, such as A431 and HaCaT, while nucleolin-negative L929 cells showed no Ce6 fluorescence (Fig. 3B). This uptake pattern can be attributed to the high-affinity binding between the AS1411 aptamer in G4DP and nucleolin, facilitating intracellular delivery. To validate this mechanism, A431 cells were pretreated with free AS1411 (0.5 μM) for 1 h to saturate the surface nucleolin, followed by treatment with G4DP. Interestingly, this pretreatment inhibited the cellular uptake of G4DP (Fig. 3C), providing evidence for the crucial role of nucleolin in cellular uptake.

Subsequently, the response of G4DP to Survivin mRNA was evaluated in A431 cells. Cells were treated with G4DP (with or without BHQ-1) for different time points to monitor the response dynamics to intracellular Survivin. Without the quencher, evident Ce6 fluorescence was observed within the cells at 8 h (Fig. 3D), gradually increasing over 24 h, indicating the intracellular delivery kinetics of G4DP. In contrast, with the presence of the quencher, fluorescence activation occurred at a much slower rate and was observed at 24 h. This delay is likely attributed to the process of Survivin mRNA response. To further confirm this, the expression level of Survivin was depleted using small interfering RNA (siRNA) (Fig. 3E). Consistently, Survivin depletion significantly reduced the Ce6 fluorescence in A431 cells (Fig. 3F and G).

Taken together, these results demonstrate that the activation of G4DP is determined by 2 factors: primary targeted uptake mediated by AS1411 and secondary response triggered by Survivin mRNA. This “AND” logic further enhances the specificity toward nucleolin-positive and Survivin-overexpressing tumor cells.

### Selective PDT toward tumor cells with enhanced efficacy via Survivin silencing and oxygenation

**Fig. 4.**
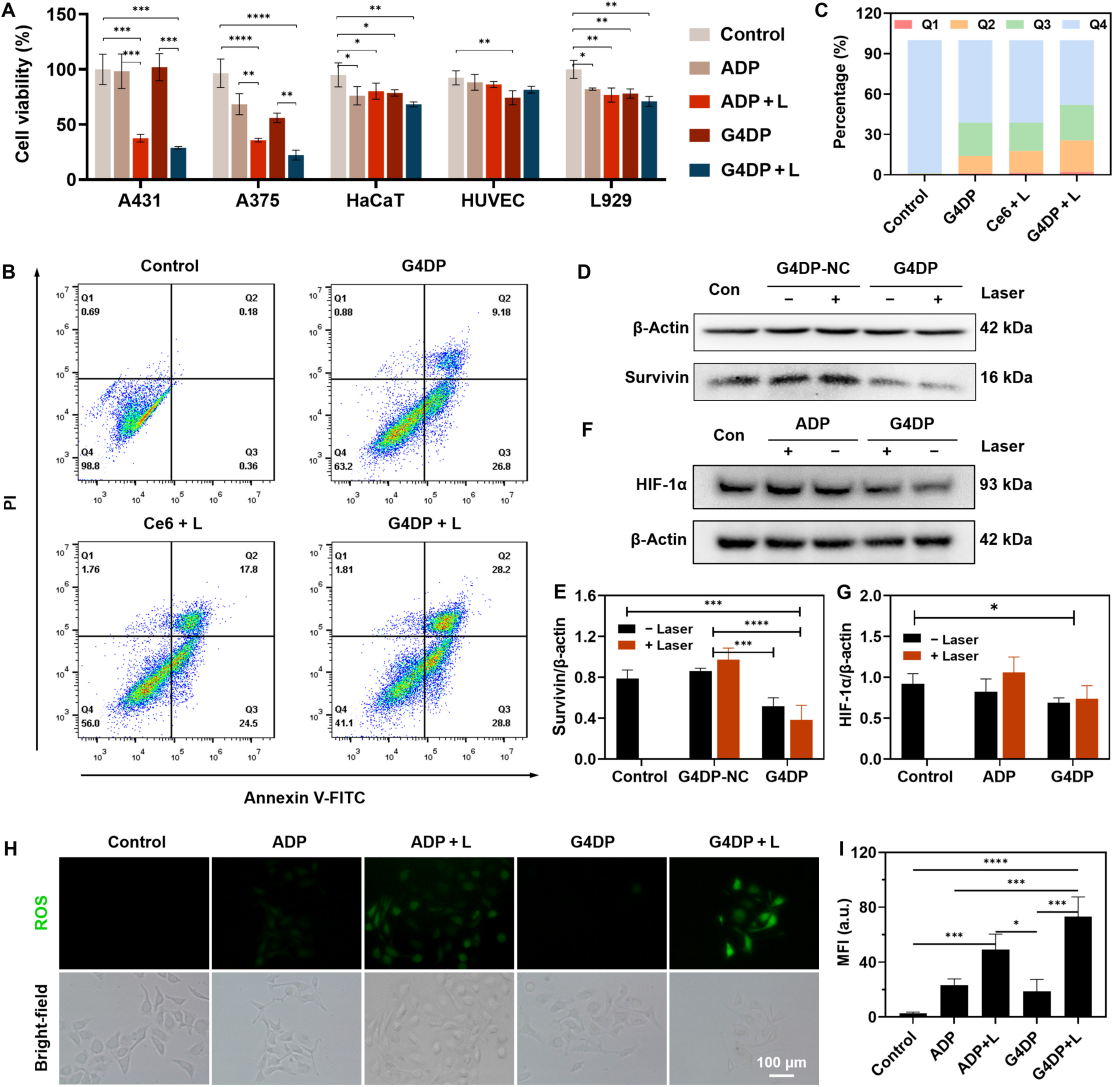
Selective PDT toward tumor cells with enhanced efficacy via Survivin silencing and oxygenation. (A) Cell viability of different cell lines after different treatments. L, laser irradiation. (B) Flow cytometry analysis of A431 cells after different treatments and (C) percentage of cells in different states: Q1 – necrosis, Q2 – late apoptosis, Q3 – early apoptosis, Q4 – live cells. (D) Western blot analysis of Survivin expression in A431 cells after various treatments, and (E) relative quantification of protein levels. (F) Western blot analysis of HIF-1α expression in A431 cells after various treatments and (G) relative quantification of protein levels. (H) Fluorescence imaging of ROS by DCFH-DA staining in A431 cells with various treatments, scale bar = 100 μm, and (I) intensity quantification of ROS levels. *P < 0.05, **P < 0.01, ***P < 0.001, and ****P < 0.0001.

Following confirmation of internalization into tumor cells, the in vitro antitumor effect of G4DP was assessed using an MTT assay. The viability of skin cancer cell lines (A431 and A375) and normal cell lines (HaCaT, HUVEC, and L929) was evaluated. In the absence of a laser, both ADP and G4DP did not cause high cytotoxicity toward all types of cells (Fig. 4A), indicating high biocompatibility. Upon laser irradiation, significant inhibition of viability of tumor cells was observed, but without any apparent toxicity to normal cells. Specifically, the viability of cancer cell lines decreased to less than 30%, while the normal cells maintained viability at 70% to 80%. These results demonstrated the selective activation of the PDT effect by both ADP and G4DP in tumor cells, leading to cytotoxicity. Interestingly, G4DP exhibited relatively stronger antitumor effects, likely due to the catalytic activity of G4/hemin DNAzyme within G4DP, providing a self-oxygen supply, as demonstrated below.

Apoptosis of A431 cells was measured after various treatments. Notably, G4DP induced cellular apoptosis to a certain degree even in the absence of laser irradiation (Fig. 4B and C). This phenomenon may be attributed to the combined impact of Survivin ASO (as further described below) and AS1411. Importantly, our supplementary data in Fig. S4A corroborates that AS1411 could induce early apoptosis. Laser irradiation, combined with free Ce6, effectively induced cell apoptosis through the photodynamic effect, resulting in a late apoptosis rate of 17.8%. After treatment with G4DP, the apoptosis rates further increased to 57.0%, attributable to that AS1411 aptamer facilitates heightened cellular uptake of G4DP by the cells (Fig. S4B). What is more, the gene-silencing effect of Survivin ASO and the catalase-like activity of G4/hemin also play crucial roles in cytotoxicity of G4DP for tumor cells.

To confirm these mechanisms, the contributions of ASO and DNAzyme were investigated separately. Survivin, an inhibitor of apoptosis protein, is detected in tumor and fetal tissues but is absent in most adult differentiated tissues [39,40]. Survivin levels in tumors are inversely correlated with patient prognosis, and up-regulation of Survivin is associated with resistance to chemotherapy, radiotherapy, and PDT [16,41]. In this study, a Survivin antisense nucleotide (DNA strand #2) was designed to hybridize with strand #1, forming G4DP. As a control, a nonsense DNA sequence was designed to hybridize with strand #1, resulting in G4DP-NC. Compared to G4DP-NC, G4DP remarkably reduced Survivin expression both in the absence and presence of laser irradiation (Fig. 4D and E), demonstrating its capability to silence the Survivin gene.

To evaluate the role of G4/hemin in cellular oxygenation, the expression level of intracellular HIF-1α, an oxygen-sensing transcription factor, was indirectly assessed. Hypoxia in the tumor microenvironment induces HIF-1α expression and contributes to tumor resistance to PDT. Therefore, increasing cellular oxygen concentration can inhibit HIF-1α expression and enhance PDT efficacy. Under laser irradiation, ADP enhanced HIF-1α expression (Fig. 4F and G), potentially due to Ce6-mediated PDT depleting intracellular oxygen and inducing HIF-1α expression. In contrast, G4DP significantly reduced HIF-1α expression even under laser irradiation, indicating that the catalase-like activity of G4/hemin DNAzyme could increase cellular oxygen content.

Furthermore, intracellular levels of reactive oxygen species (ROS) were assessed using a DCFH-DA probe to evaluate the efficiency of G4/hemin in synergistic ROS production with Ce6. Without any treatment, minimal fluorescence corresponding to basal ROS levels was observed in A431 cells. Upon irradiation, both ADP and G4DP groups exhibited significant fluorescence intensity, indicating their capability to generate ROS through the photodynamic effect. Interestingly, after treatment with G4DP, cells displayed brighter fluorescence compared to the ADP group, attributed to the catalase-like activity of G4/hemin increasing cellular oxygen content (Fig. 4H and I). Therefore, G4DP could selectively damage tumor cells, and its PDT effect could be enhanced via the synergistic effect of Survivin ASO and the catalase-like activity of G4/hemin.

### Targeting delivery and accurate activation of G4DP in tumors with decrease of phototoxicity in vivo

**Fig. 5.**
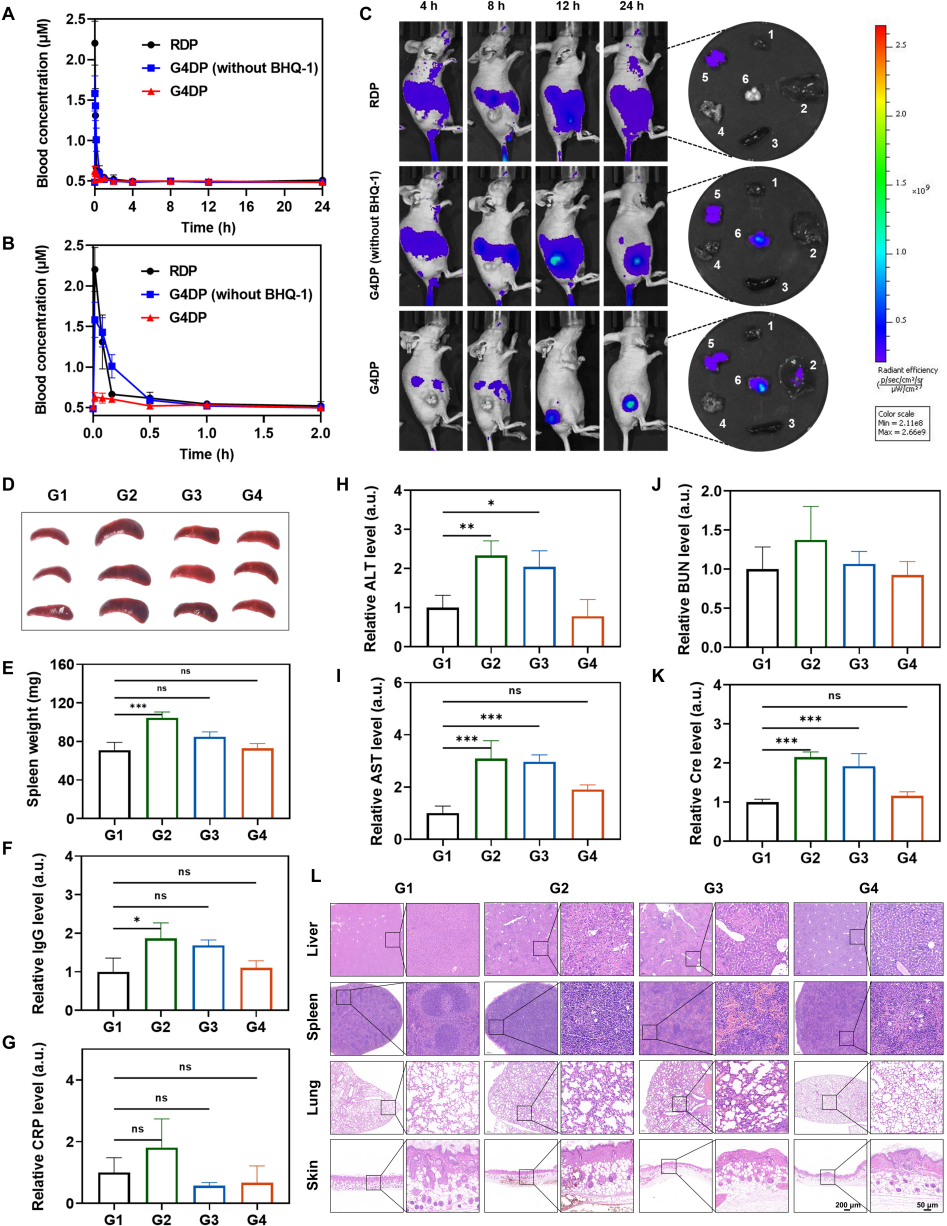
Targeting delivery and accurate activation of G4DP in tumors with decrease of phototoxicity in vivo. (A) The plasma Cy5.5 concentration–time profile within 24 h after intravenous injection of different treatments. (B) The plasma Cy5.5 concentration–time profile within 2 h magnified from (A). (C) The fluorescence images of mice taken at 4, 8, 12, and 24 h post intravenous injection of different treatments. (D) Photos of spleen harvested from mice in each group at 24 h post intravenous injection. (E) Spleen weight after different treatments. (F) Relative IgG levels in the mice after different treatments. (G) Relative CRP levels in the mice after different treatments. The blood biochemical indexes of (H) ALT, (I) AST, (J) BUN, and (K) Cre. (L) The images of H&E staining for different tissues after different treatments. Scale bar = 200 and 50 μm, respectively. G1, con; G2, RDP; G3, G4DP (without BHQ-1); and G4, G4DP. *P < 0.05, **P < 0.01, ***P < 0.001, and ****P < 0.0001.

The in vivo performance of G4DP was investigated using Cy5.5 fluorophore as a substitute for Ce6 to enable convenient monitoring. Upon intravenous injection, blood samples were collected at various time points, and the fluorescence was quantified. A control group, denoted as RDP, was implemented by substituting the AS1411 fragment in G4DP with a random sequence of equal length, excluding the quencher (BHQ-1). Subsequent analysis indicated a swift decrease in RDP concentration within the mice’s bloodstream (Fig. 5A and B), underscoring the rapid distribution and metabolic processing of RDP in vivo. In contrast, the modified G-quadruplet structure of G4DP (lacking BHQ-1) exhibited a notably slower decrease in blood concentration. This prolonged retention could be attributed to the influence of nucleolin protein present on vascular endothelial cells, potentially affecting the distribution pattern of G4DP. Furthermore, minimal fluorescence was observed in the blood, indicative of the stable hybridization status of the structure in its “off” mode. This observation aligns with the in vitro stability results. The stable “off” mode maintained during in vivo circulation is critical in mitigating the phototoxic potential of G4DP.

The in vivo biodistribution was explored using a subcutaneous xenograft tumor model in BALB/c nude mice when the tumor size reached approximately 100 mm^3^. G4DP without quencher exhibited rapid distribution in vivo, and importantly, a gradual tumor accumulation was observed at 12 and 24 h, indicating the targetability of the structure in vivo. This tumor-targeting effect can be attributed to the AS1411 aptamer, which binds to nucleolin that is overexpressed on the surface of tumor cells. In this case, broad fluorescence was observed in the mice body, while minimal fluorescence was seen in the tumor, indicating poor tumor targetability. Additionally, strong fluorescence was observed in the liver for both G4DP and RDP, indicating major liver metabolism. However, G4DP exhibited weak fluorescence in the body, confirming the “off” mode of the structure during in vivo circulation. Over an extended circulation time, the fluorescence gradually lit up specifically in the tumor, demonstrating its selective activation in tumor cells triggered by Survivin mRNA. Consistently, ex vivo imaging of tumor tissue showed strong fluorescence for G4DP (Fig. 5C). These results demonstrated that G4DP can effectively accumulate in the tumor and be selectively activated, allowing for efficient and targeted PDT while minimizing side effects.

To directly evaluate the advantage of G4DP in decreasing phototoxicity, tumor-bearing mice were exposed to light (light-emitting diode 630 nm, 10 mW/cm^2^) for 1 h at 4 h post intravenous injection to mimic accidental light exposure, and pathological analyses were performed. Severe phototoxic reactions were detected in mice injected with RDP without quencher (Fig. 5D to K), presenting as an enlarged spleen, increased allergy markers (IgG and C reaction protein, etc.), and liver/kidney function markers (AST, ALT, Cre, and BUN). G4DP without quencher also induced severe phototoxic responses, but these were generally lower than the RDP group, likely due to its tumor targetability. In contrast, G4DP substantially reduced Ce6 phototoxicity, with all parameters within normal levels. H&E staining of liver, lung, and spleen tissues of mice treated with RDP or G4DP without quencher showed different degrees of inflammatory cell infiltration and pathological structural changes, while G4DP-treated mice did not show any symptoms, confirming the decreased side effects of G4DP for PDT (Fig. 5L and H&E staining of the other main organs showed in Fig. S5A).

Since PDT is usually administered via local skin therapy, we also mimicked this situation by subcutaneous injection of G4DP, followed by local irradiation at the administration site for 10 min (630 nm, 100 mW/cm^2^). Substantial local skin damage, such as redness, thickening of the epidermis, and destruction of the skin structure, was observed in mice treated with RDP or G4DP without quencher. However, there was no substantial change in mice treated with G4DP (Fig. 5L and Fig. S5B). These results strongly indicate that G4DP could effectively reduce the potential phototoxic side effects of Ce6.

### Antitumor effect of G4DP in xenograft tumor-bearing mice model

**Fig. 6.**
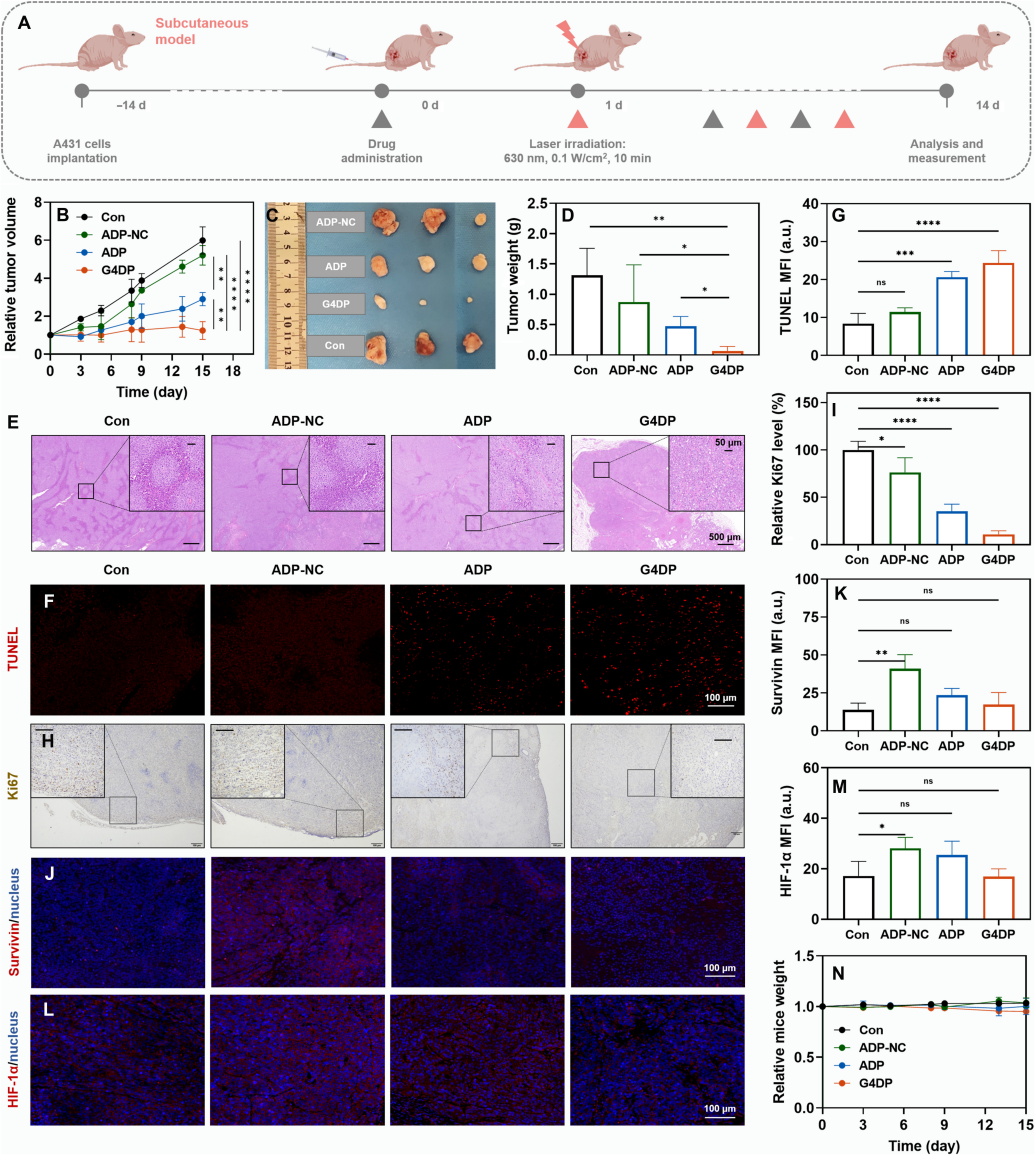
Antitumor effect of G4DP in xenograft tumor-bearing mice model. (A) Schematic depiction of the experimental approach for the antitumor treatment. (B) Tumor growth curves of different groups. (C) Photos of tumors harvested from mice in each group on day 14. (D) Tumor weight after various treatments. (E) Images of H&E staining for tumors after different treatments. Scale bar = 500 and 50 μm (inset), respectively. (F) Immunofluorescence staining of TUNEL (red) in tumor tissue and (G) the mean fluorescence intensity results. Scale bar = 100 μm. (H) Immunohistochemical staining of Ki67 in tumor tissue and (I) the relative quantified results. Scale bar = 100 and 50 μm (inset), respectively. (J) Immunofluorescence staining of Survivin (red) in tumor tissue and (K) the mean fluorescence intensity results. Scale bar = 100 μm. (L) Immunofluorescence staining of HIF-1α (red) in tumor tissue and (M) the mean fluorescence intensity results. Scale bar = 100 μm. (N) The relative mice weight of mice after different treatments. *P < 0.05, **P < 0.01, ***P < 0.001, and ****P < 0.0001.

The in vivo antitumor effect of G4DP was assessed using a subcutaneous xenograft tumor-bearing model in BALB/c nude mice. When the tumor volume grew to a size of approximately 50 mm^3^, G4DP was intravenously injected every 3 d for a total of 3 doses (Fig. 6A). At 24 h postinjection, irradiation was performed at the tumor site for 10 min (630 nm, 100 mW/cm^2^). As controls, ADP (excluding the contribution of G4/hemin DNAzyme) and ADP with a random ASO sequence that cannot silence the Survivin gene (termed ADP-NC, excluding the contribution of both G4/hemin DNAzyme, ASO, and quencher) were also administered.

Interestingly, ADP-NC did not show an obvious therapeutic effect compared to the PBS control (Fig. 6B). This was consistent with previous reports that tumor cells can develop various mechanisms to resist PDT [4,13,16]. In contrast, ADP exhibited a stronger antitumor efficacy, which can be attributed to the combination of PDT and the gene-silencing effect of anti-Survivin ASO, highlighting the role of Survivin in tumor cell Survival in response to PDT. Among the treatments, the most substantial antitumor efficacy was observed in the G4DP group, completely suppressing tumor growth. Therefore, the integration of G4/hemin DNAzyme further invigorated PDT via in situ oxygen generation. After 14 d of treatment, the mice were sacrificed, and the tumor tissues were collected for further analysis. Based on tumor weight, the tumor suppression effect was ranked in order of G4DP > ADP > ADP-NC (Fig. 6C and D), highly consistent with in vivo monitoring. Histological examination of tumor tissues revealed that G4DP induced the highest level of tumor destruction, showing substantial karyopyknosis and cavitation based on H&E staining (Fig. 6E), as well as the strongest cell apoptosis according to TUNEL and Ki67 staining (Fig. 6F to I).

The antitumor mechanisms of G4DP were further explored in vivo. Immunofluorescent assays were used to visualize the Survivin protein level. The ADP-NC group exhibited a higher Survivin level in tumor tissue than the control group, indicating up-regulation of Survivin in response to PDT. Upon addition of Survivin ASO, both ADP and G4DP strongly down-regulated Survivin, promoting PDT-induced apoptosis (Fig. 6J and K). Another important aspect of this functional DNA structure is the incorporation of G4/hemin DNAzyme for catalytic oxygenation. The expression of HIF-1α was measured as an indicator of hypoxia. Both ADP-NC and ADP-treated mice showed increased levels of HIF-1α compared to the control group, indicating that Ce6-mediated PDT consumes O_2_ at the tumor site and aggravates tumor hypoxia. In contrast, G4DP effectively down-regulated the expression of HIF-1α due to the catalase-like activity of G4/hemin (Fig. 6L and M). These results suggest that G4DP may synergistically enhance PDT by simultaneously down-regulating Survivin expression and ameliorating hypoxia via catalytic oxygenation.

Meanwhile, the biosafety of each treatment was evaluated. The body weight of all mice remained unchanged, indicating their good body condition during treatment (Fig. 6N). After treatment, blood was collected for biochemical analysis, including AST, ALT, Cre, and BUN. Except for the ADP-NC group, which caused a slight abnormal increase in the indicators, the other groups showed no substantial differences from the control group (Fig. S6). These results confirm the high biocompatibility of G4DP for in vivo applications.

### Antitumor effect of G4DP in PDX model

Encouraged by the robust therapeutic efficacy of G4DP in the subcutaneous xenograft tumor model, we sought to investigate its potential applicability in conquering PDX models, which hold greater clinical relevance as they consist of tumor cells derived directly from cancer patients. PDX models have been shown to retain the biological characteristics of the parental tumors, making them valuable preclinical models for assessing the in vivo antitumor effect of drugs [33,42]. Accordingly, we established the PDX SCC model to further evaluate the antitumor potential of G4DP (Fig. S7).

**Fig. 7.**
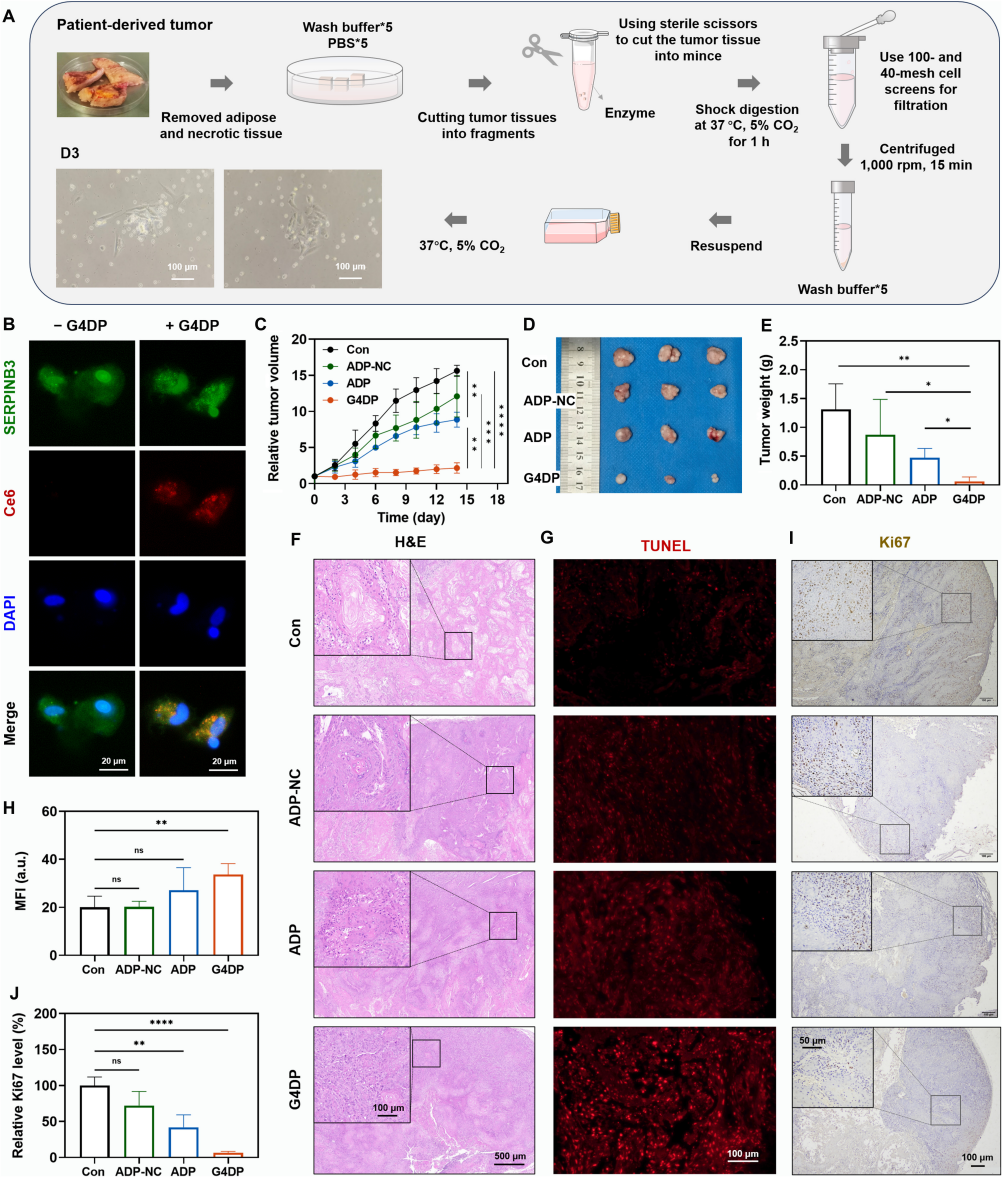
Antitumor effect of G4DP in patient-derived xenografts model. (A) Diagram of the establishment of primary SCC cells. (B) Immunofluorescence staining of SERPINB3 (green) and the uptake and activation of G4DP in primary SCC cells. Scale bar = 20 μm. (C) Tumor-growth curves of different groups. (D) Photos of tumors harvested from mice in each group on day 14. (E) Tumor weight after various treatments. (F) The images of H&E staining for tumors after different treatments. Scale bar = 500 and 100 μm (inset), respectively. (G) Immunofluorescence staining of TUNEL (red) in tumor tissue and (H) the mean fluorescence intensity results. Scale bar = 100 μm. (I) Immunohistochemical staining of Ki67 in tumor tissue and (J) the relative quantified results. Scale bar = 100 and 50 μm (inset), respectively. *P < 0.05, **P < 0.01, ***P < 0.001, and ****P < 0.0001.

To verify its targetability, we first cultured primary squamous cells from patient-derived tumors (Fig. 7A). The extracted primary cells comprised both tumor cells and normal cells, such as fibroblasts, displaying distinct cell morphologies (Fig. 7A, inset). To specifically visualize the tumor cells, we stained the cells with SERPINB3 (squamous cell carcinoma antigen, SCCA1), a serine protease inhibitor highly expressed in various SCCs and a specific diagnostic marker for SCC [43,44]. Fluorescence images revealed the presence of both SERPINB3-negative and SERPINB3-positive cells, indicating the coexistence of SCC and nontumor cells in the cell extract (Fig. S8). Subsequently, G4DP was added for coincubation to evaluate its response to different cell types. Encouragingly, G4DP with red fluorescence selectively appeared in SERPINB3-positive SCC cells but not in SERPINB3-negative cells (Fig. 7B and Fig. S8), confirming its selective activation for primary SCC cells.

We then proceeded to evaluate the antitumor effect on the PDX SCC model as previously described earlier. Tumor growth was dynamically monitored during treatments, and tumor tissue was extracted posttreatment for direct observation and further analysis. Overall, the tumor suppression efficacy in both the PDX SCC model and the subcutaneous xenograft tumor model exhibited a similar trend, with G4DP demonstrating the most robust efficacy, leading to complete suppression of the PDX SCC model (Fig. 7C to E). However, in this case, the ADP group showed lower efficacy compared to the subcutaneous xenograft tumor model. This discrepancy may be attributed to the PDX SCC model’s compact and highly deteriorated tumor characteristics, including evident features such as keratin pearls and intercellular bridges, resulting in more pronounced tumor hypoxia and limited PDT efficacy (Fig. 7F). Histological examinations further validated the efficacy, as evidenced by extensive tumor tissue destruction and low levels of deterioration in the G4DP group, while the ADP and ADP-NC groups displayed moderate damage (Fig. 7F). Additionally, the TUNEL assay and Ki67 staining corroborated the antitumor effect, with G4DP inducing significant tumor cell apoptosis and reduced proliferation levels, while ADP and ADP-NC exhibited moderate effects (Fig. 7G to J). These findings highlight G4DP’s selective targeting capability and efficacy in combating PDX SCC models, underscoring its potential for clinical applications.

### Biosafety evaluation of G4DP

**Fig. 8.**
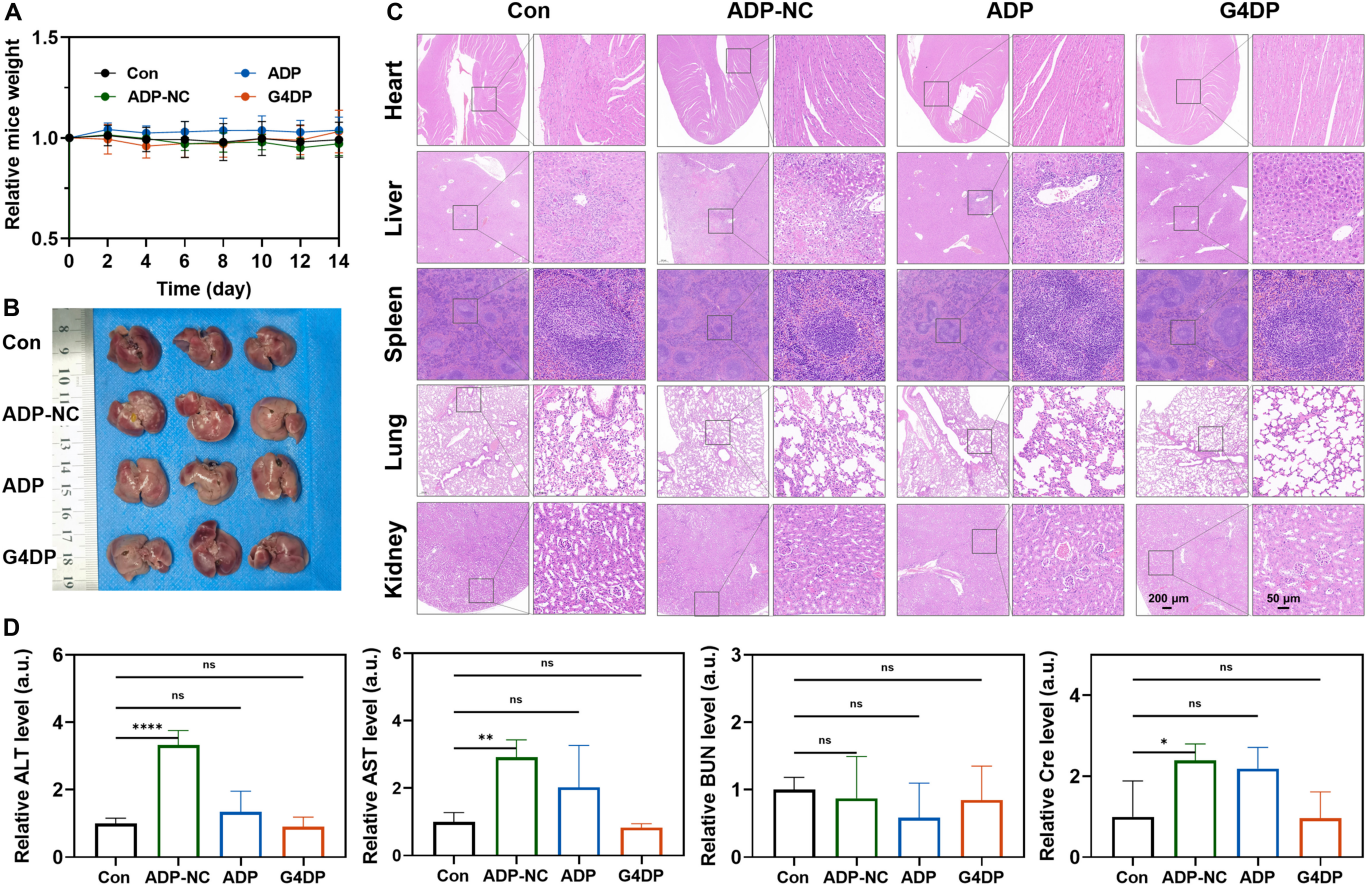
Biosafety evaluation of G4DP. (A) The relative mice weight of mice after different treatments. (B) Photos of livers harvested from mice in each group on day 14. (C) The images of H&E staining for different tissues after different treatments. Scale bar = 200 and 50 μm, respectively. (D) The blood biochemical indexes of ALT, AST, BUN, and Cre. *P < 0.05, **P < 0.01, ***P < 0.001, and ****P < 0.0001.

The safety profile of each treatment was thoroughly evaluated to assess the potential biosafety concerns of G4DP. Throughout the treatment period, the body weight of all mice remained stable, indicating their overall good physical condition (Fig. 8A). Posttreatment, major organs, including the heart, liver, spleen, lung, and kidney, were harvested for observation and subjected to histological examination using H&E staining. Notably, liver specimens from the ADP-NC-treated mice displayed white nodular lesions, indicating substantial pathological changes in this group, whereas the other treatment groups did not exhibit such changes (Fig. 8B, with a local magnification shown in Fig. S9). The H&E staining further confirmed substantial alterations in the liver tissue of ADP-NC group mice compared to the other groups. However, no substantial pathological changes were observed in the other organs, which were comparable to the control group (Fig. 8C). In addition to histological assessments, blood samples were collected for biochemical analysis, encompassing ALT, AST, BUN, and Cre. With the exception of the ADP-NC group, which exhibited a slight abnormal increase in these indicators, the other treatment groups showed no substantial differences from the control group (Fig. 8D). These findings collectively underscore the high biocompatibility of G4DP and suggest its potential for clinical translation without inducing notable systemic toxicity.

## Conclusion

In conclusion, our study introduces a novel activable PS-based therapeutic approach for SCC using the G-quadruplex/hemin-DP complex (G4DP). By employing a smart delivery system, G4DP selectively accumulated in tumor cells, responding to Survivin mRNA to trigger PDT activation. Additionally, G4DP acted as an ASO, down-regulating Survivin expression, which reversed tumor resistance to PDT. Moreover, the incorporation of hemin provided catalase activity, addressing the issue of tumor hypoxia and further enhancing PDT efficacy. In both xenograft tumor-bearing mice and PDX models, G4DP demonstrated enhanced therapeutic effects with minimized side effects, highlighting its superiority over conventional PDT strategies. The development of G4DP represents a substantial advancement in cancer nanomedicine, offering a unique and promising platform to overcome tumor resistance and enhance the selectivity of PDT while minimizing side effects. With its demonstrated potential and comprehensive characterization, G4DP emerges as an efficient and targeted therapeutic agent for cancer treatment, presenting a strong candidate for future clinical translation and cancer therapy.

## Data Availability

All data that support the findings of this study are available from the corresponding author upon reasonable request.
