# Supplementary material for: Activable Photodynamic DNA Probe with an “AND” Logic Gate for Precision Skin Cancer Therapy
Source: Research (Wash D C). 2024 Jan 24;7:0295. doi: 10.34133/research.0295 (PMC10807844; doi:10.34133/research.0295)
Supplement: Supplementary 1 — Tables S1 and S2 Figs. S1 to S7 [file research.0295.f1.zip › supporting information.docx]

Activable Photodynamic Molecular Beacon with an "AND" Logic Gate for Precision Skin Cancer Therapy

Jiaojiao Zhu^1#^, Lanyuan Peng^2#^, Shah Jehan^1, 5#^, Haiyang Wang^5^, Xiang Chen^2^, Shuang Zhao^2, 4^*, and Wenhu Zhou^1, 3^*

^1^. Xiangya School of Pharmaceutical Sciences, Central South University, Changsha, Hunan 410013, China.

^2^. Department of Dermatology, Hunan Engineering Research Center of Skin Health and Disease, Hunan Key Laboratory of Skin Cancer and Psoriasis, Xiangya Hospital, Central South University, Changsha, 410008 Hunan, China.

^3^. Key Laboratory of Biological Nanotechnology of National Health Commission, Changsha 410008, Hunan, China.

^4^. Furong Laboratory, Changsha, Hunan, China.

^5^. Department of Vascular Surgery, The First Affiliated Hospital of Guangzhou Medical University, Guangzhou, 510120, Guangdong, China.

[^#^] These authors contributed equally to this work.

Email: [zhouwenhuyaoji@163.com](mailto:zhouwenhuyaoji@163.com); [shuangxy@csu.edu.cn](mailto:shuangxy@csu.edu.cn)

**Table S1.** The DNA sequences used in this study.

**
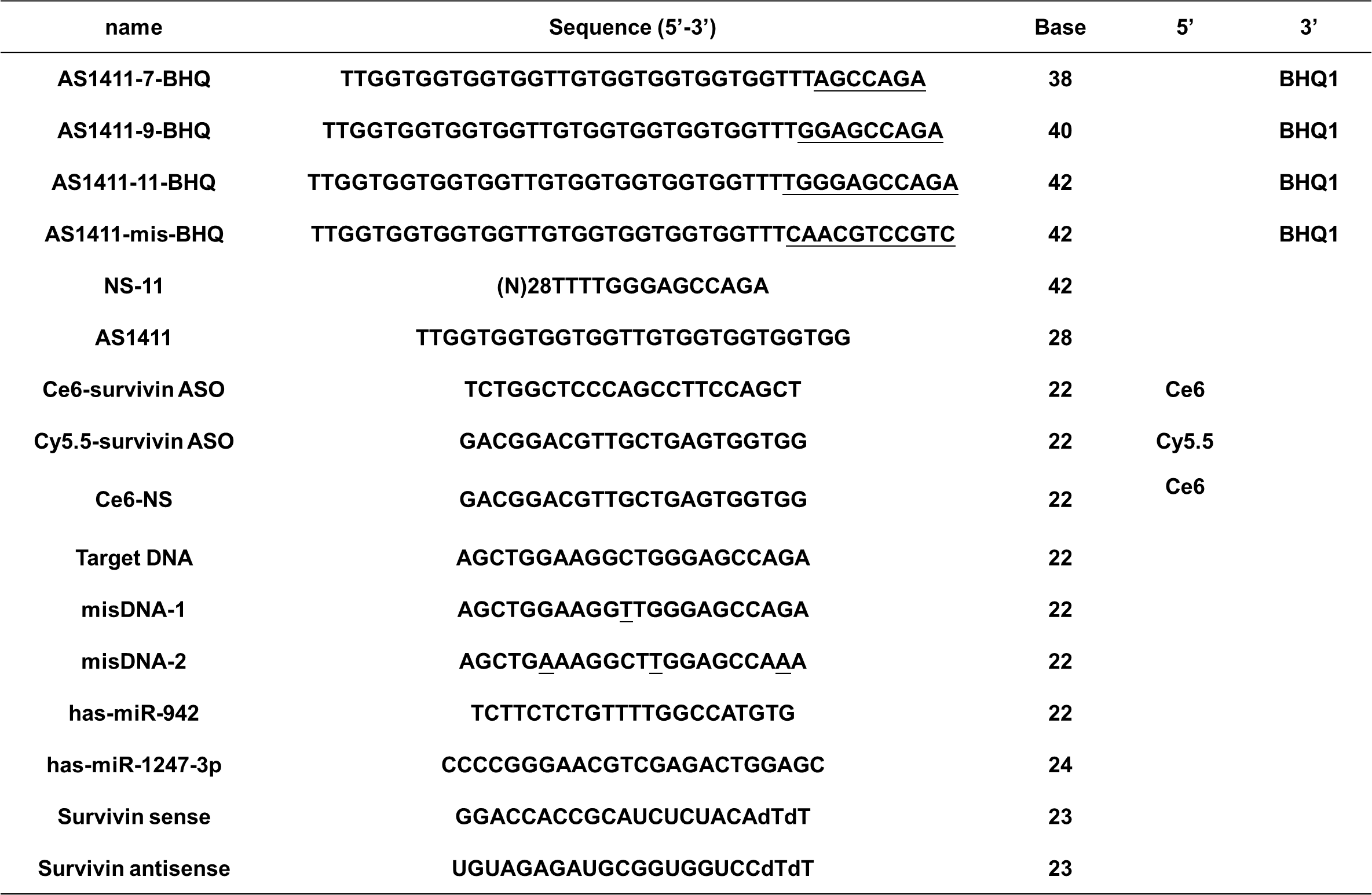
**

**Table S2.** The abbreviation of different AMBs in this study.

**
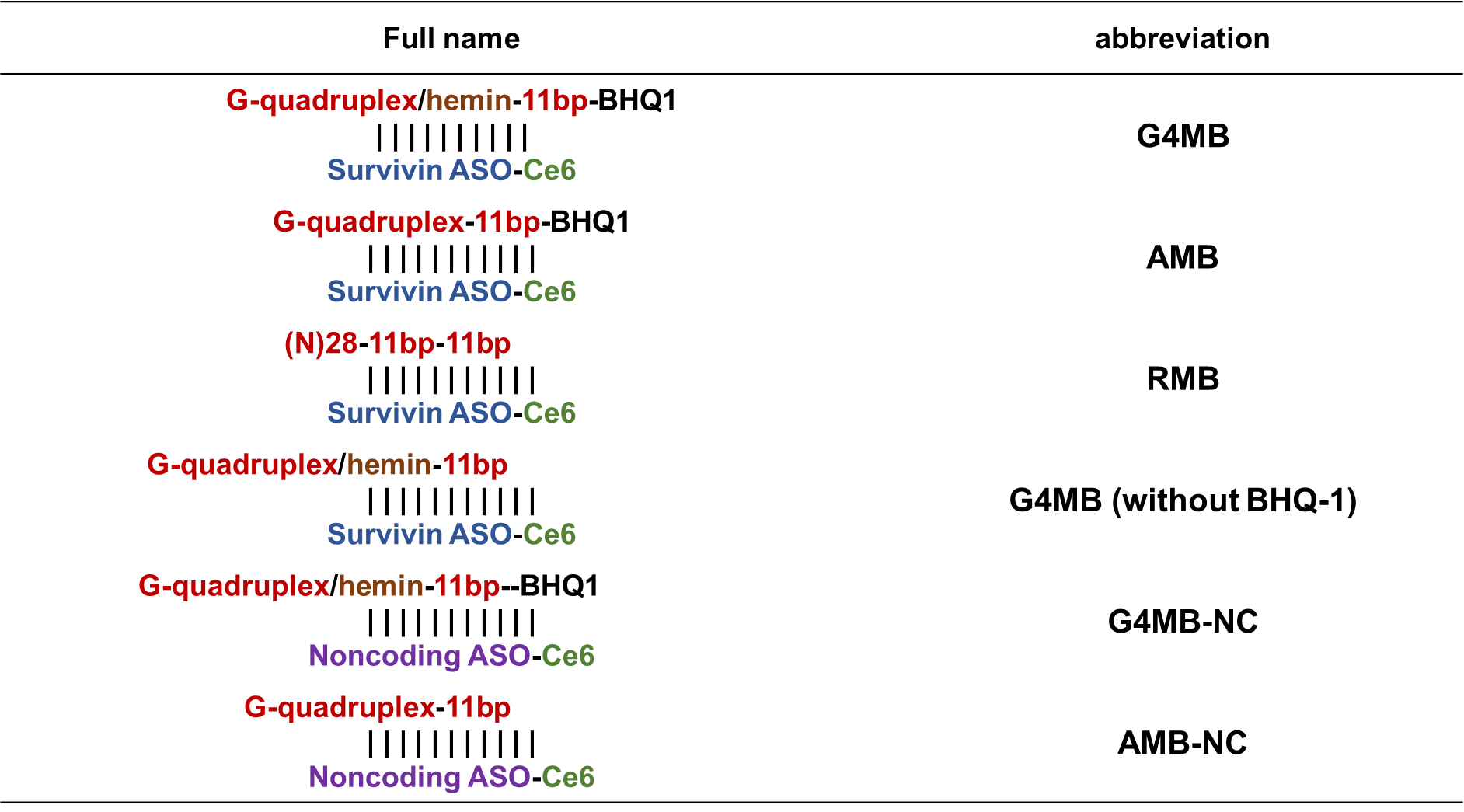
**

**
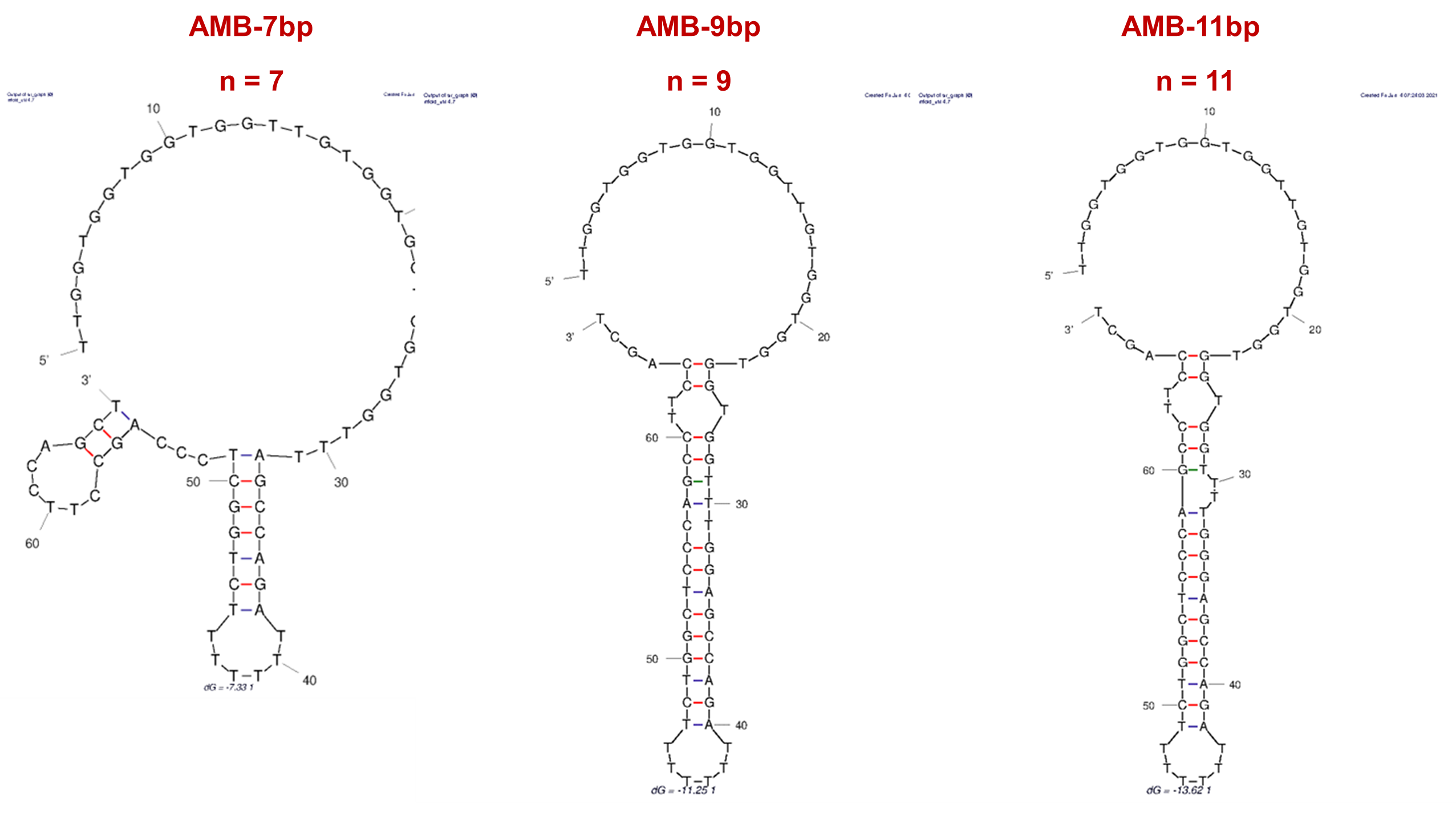
**

**Figure S1.** The secondary structure of AMBs is predicted by the Mfold web server for hybridization.

**
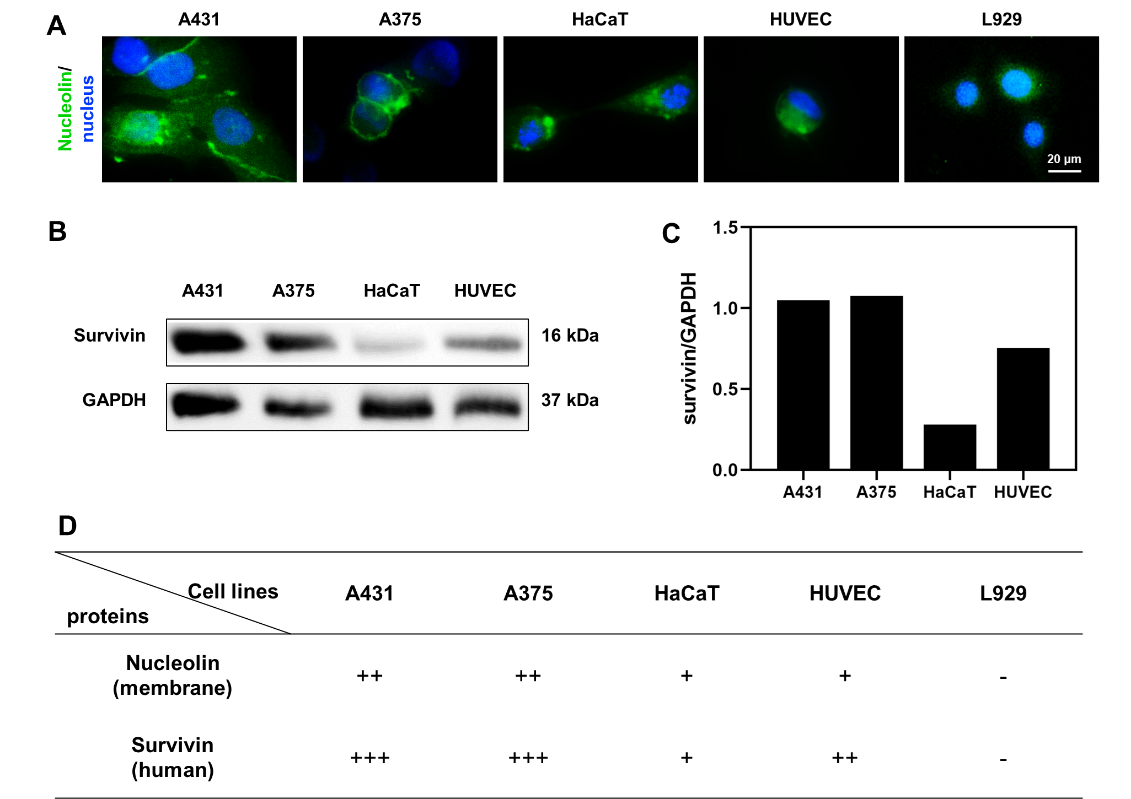
**

**Figure S2.** A) The fluorescence images show nucleolin expression in different cell lines. B) Western blot analysis of the Survivin expression in different cell lines and C) the relative quantification of protein levels. D) The degree of expression of Survivin and nucleolin in different cell lines.

**
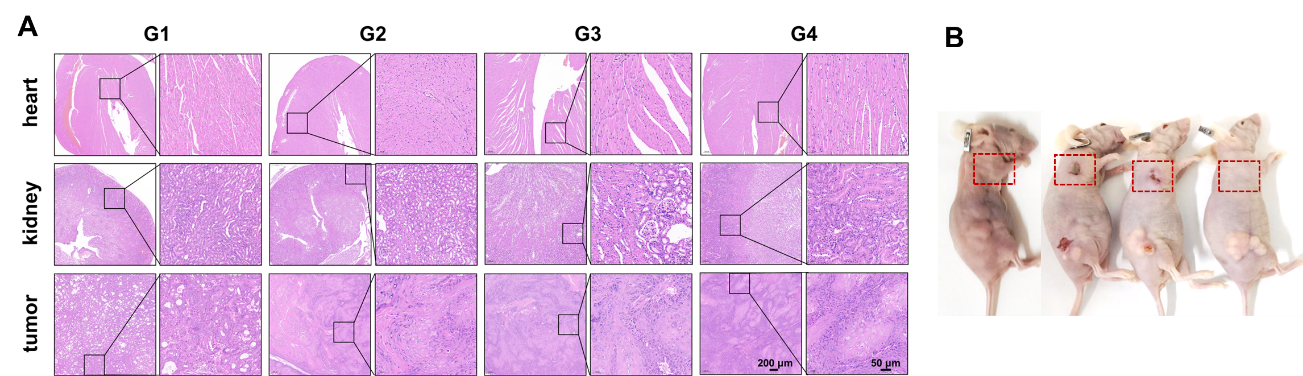
**

**Figure S3.** A) Images of H&E staining of heart, kidney, and tumor after different treatments. B) Photos of mice after different treatments. The red box shows the damage after local subcutaneous injection of different AMBs after the laser irradiation. G1: con, G2: RMB, G3: G4MB (without BHQ-1), and G4: G4MB.

**
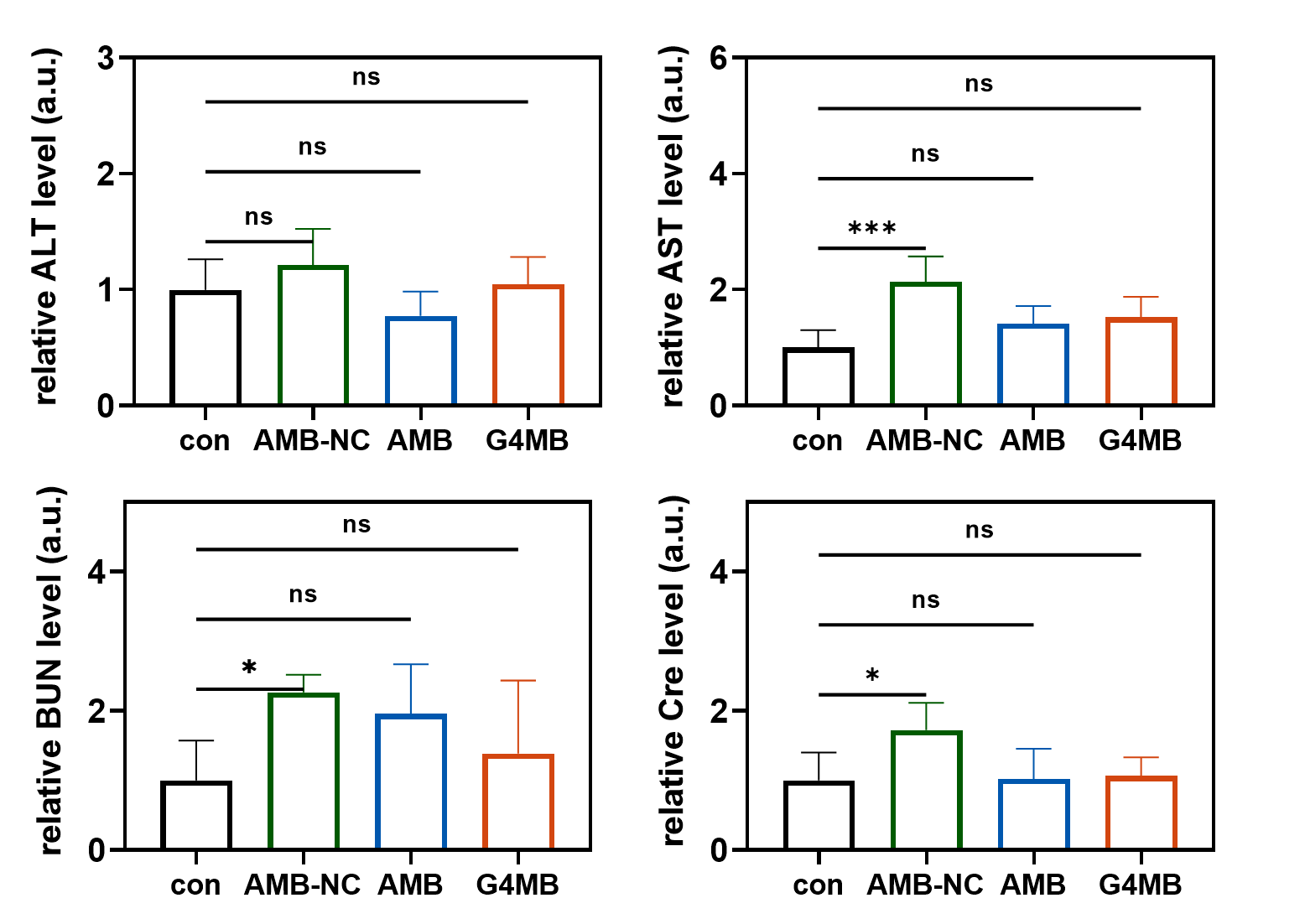
**

**Figure S4.** The blood biochemical indexes of ALT, AST, BUN, and Cre after different antitumor treatments.

**
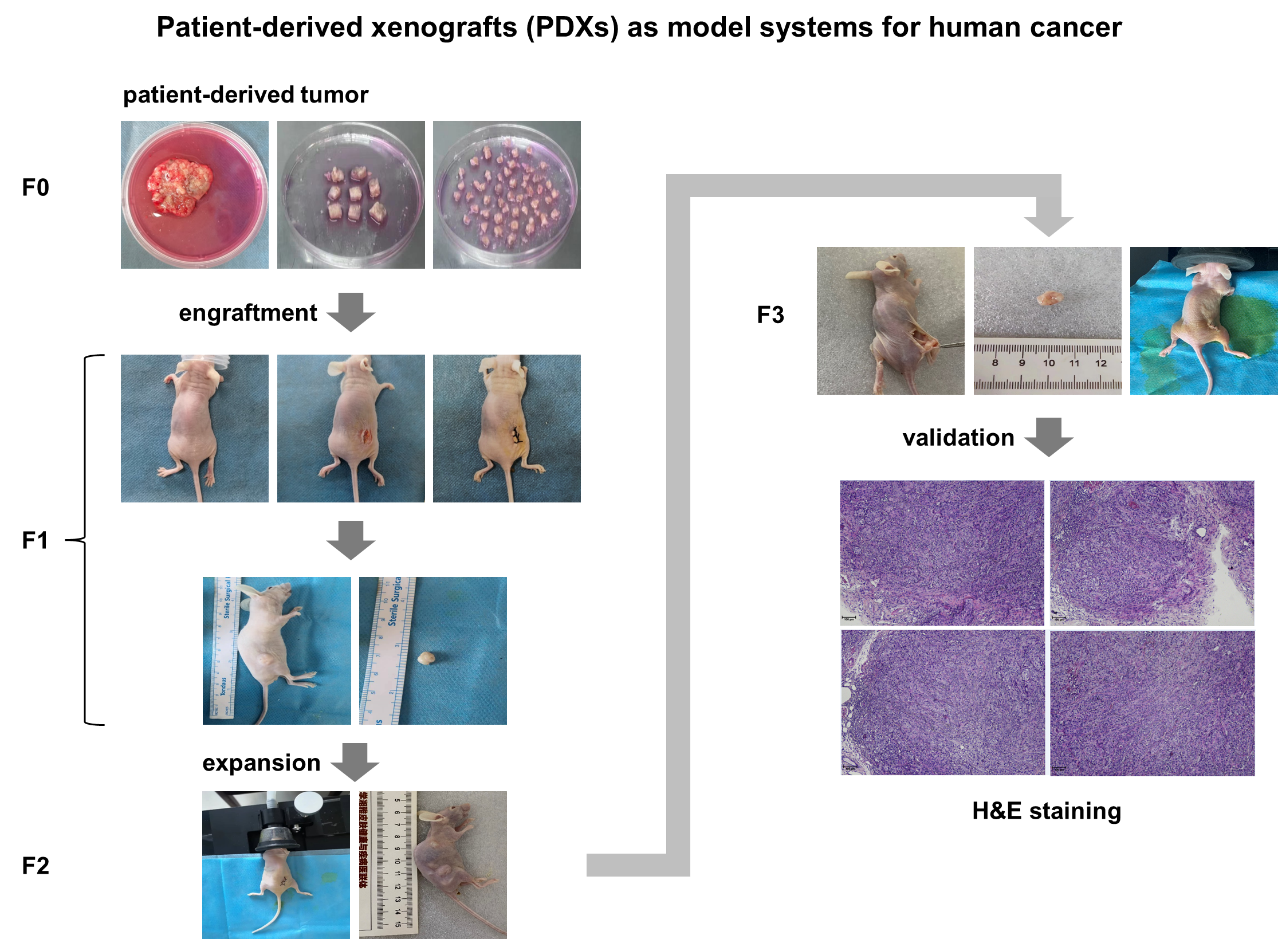
**

**Figure S5.** Diagram of the establishment of PDX SCC models.

**
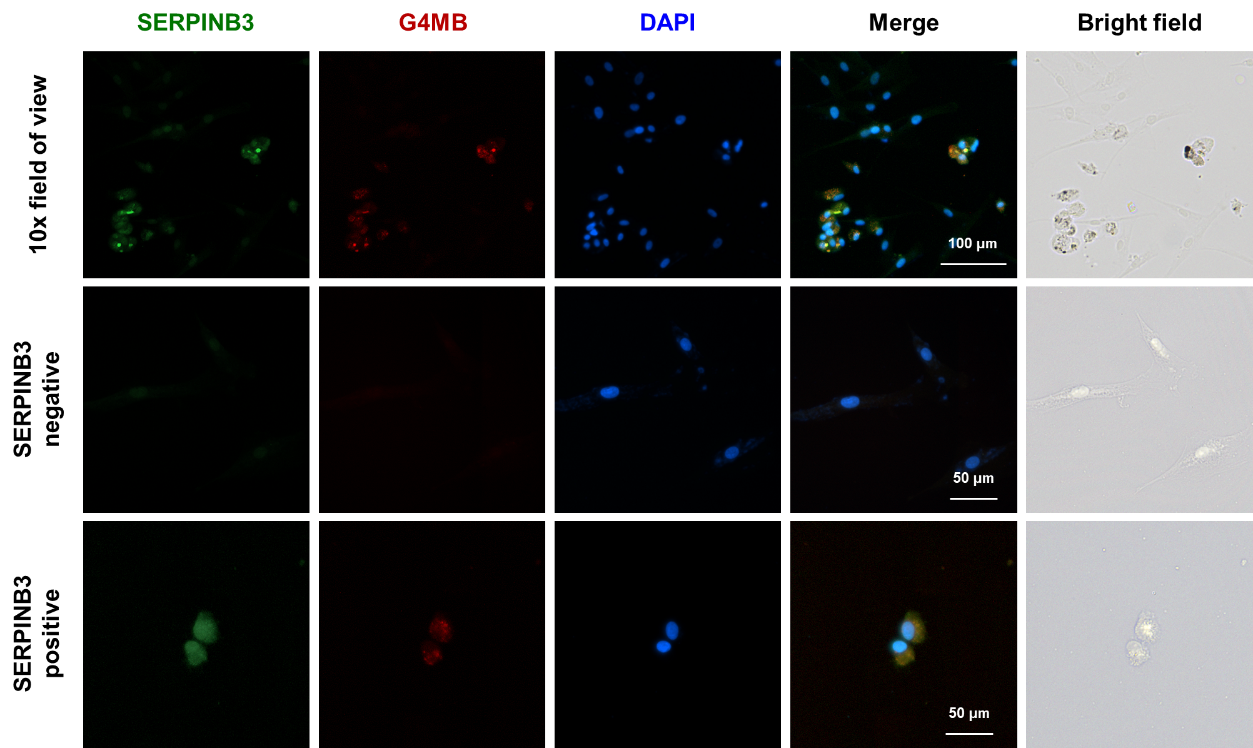
**

**Figure S6.** Immunofluorescence staining of SERPINB3 (green) and the uptake and activation of GHB-SC in primary SCC cells in a 10x field of view. Scale bar = 100 μm and 50 μm, respectively.

**
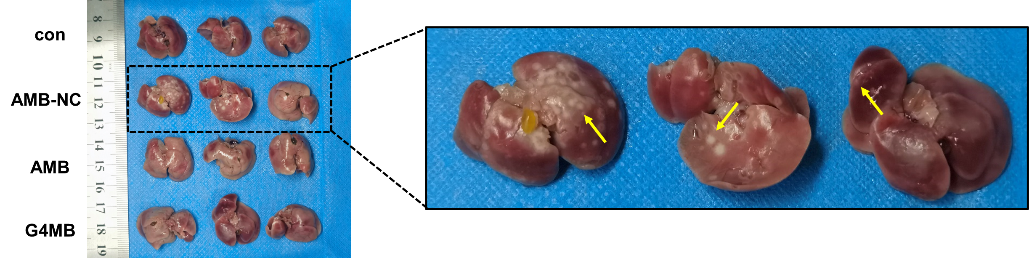
**

**Figure S7.** Photos of livers harvested from mice in each group on day 14. On the left is an enlarged view of the liver in the AMB-NC group. The yellow scissors indicate white nodular lesions.
