# Supplementary figures and images for: Activable Photodynamic DNA Probe with an “AND” Logic Gate for Precision Skin Cancer Therapy
Source: Research (Wash D C). 2024 Jan 24;7:0295. doi: 10.34133/research.0295 (PMC10807844; doi:10.34133/research.0295)

## ADP-7bp

n = 7

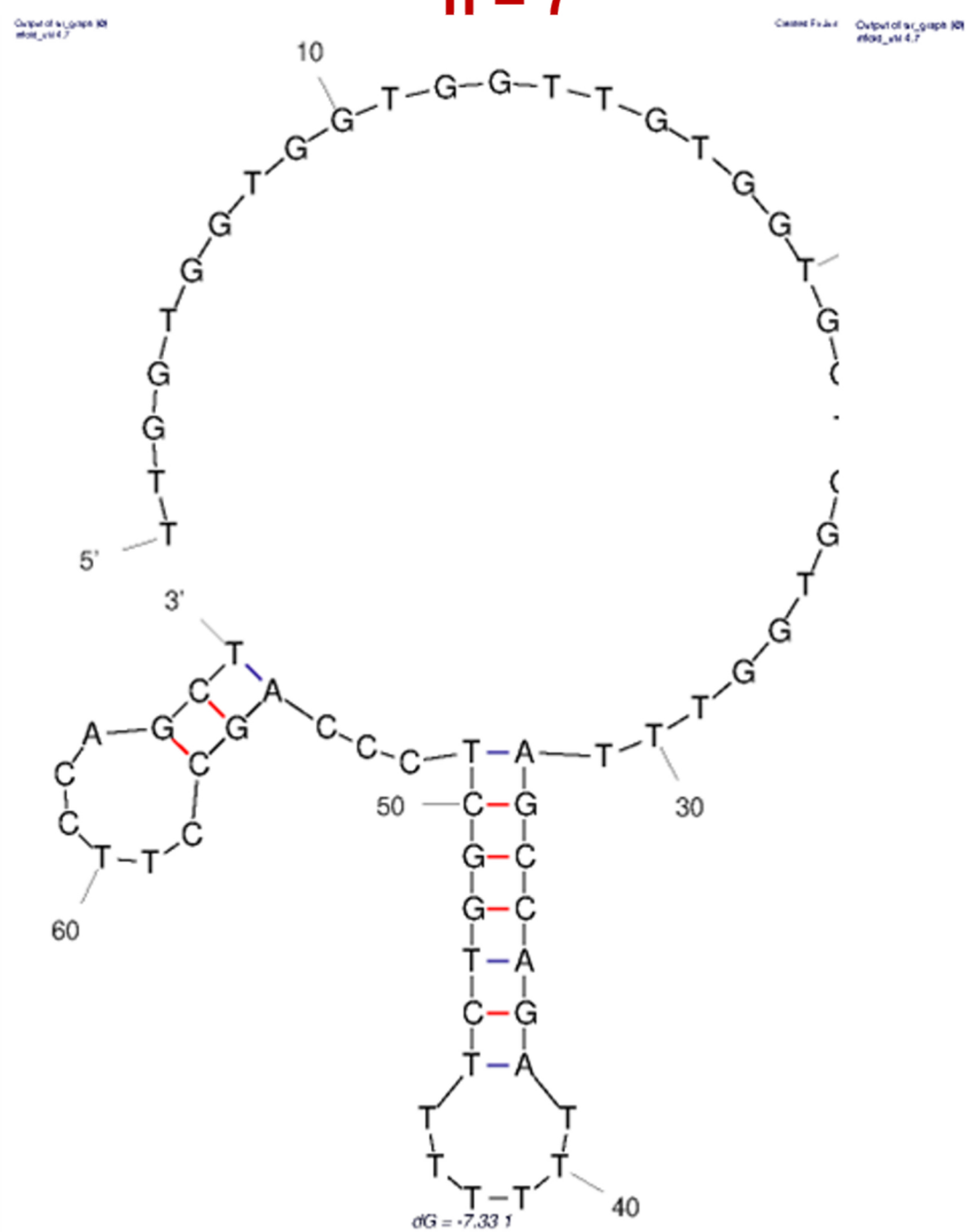

## ADP-9bp

n = 9

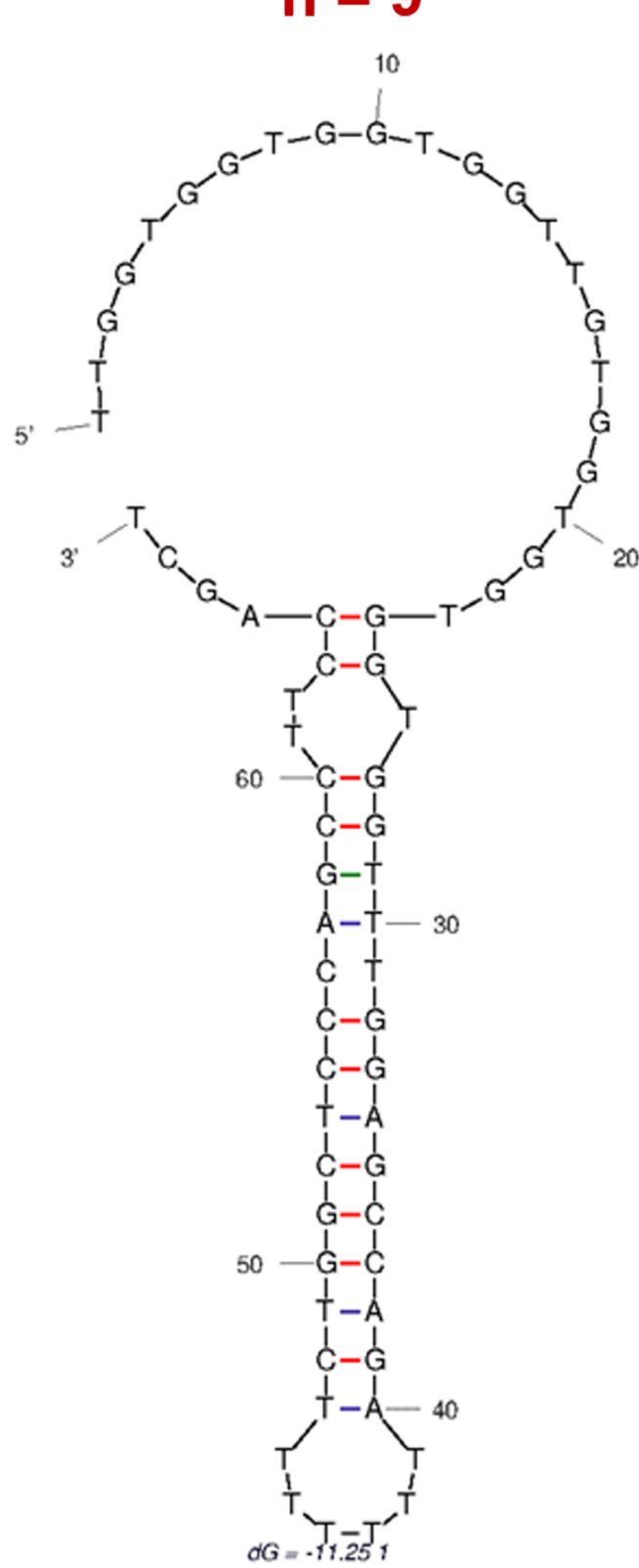

## ADP-11bp

n = 11

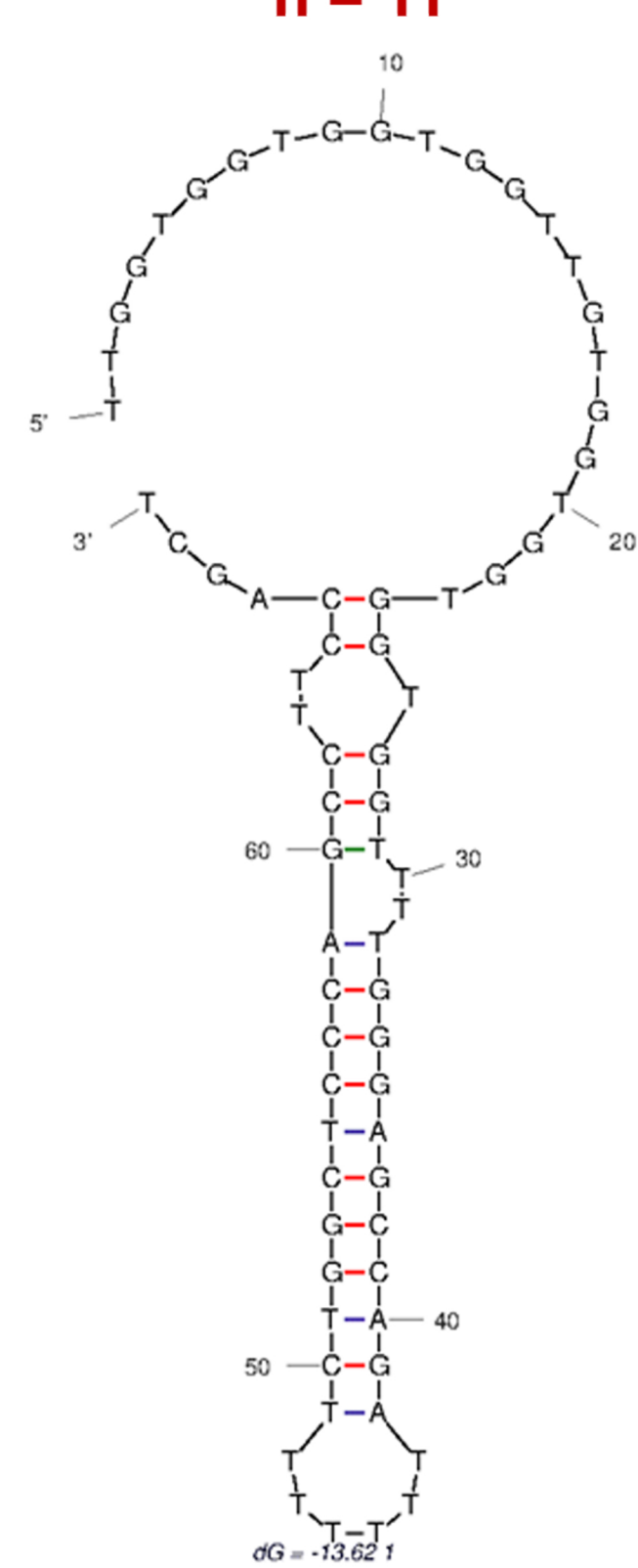

Supplement: Supplementary 1 — Tables S1 and S2 Figs. S1 to S7 [file research.0295.f1.zip › Figure S1.pdf]

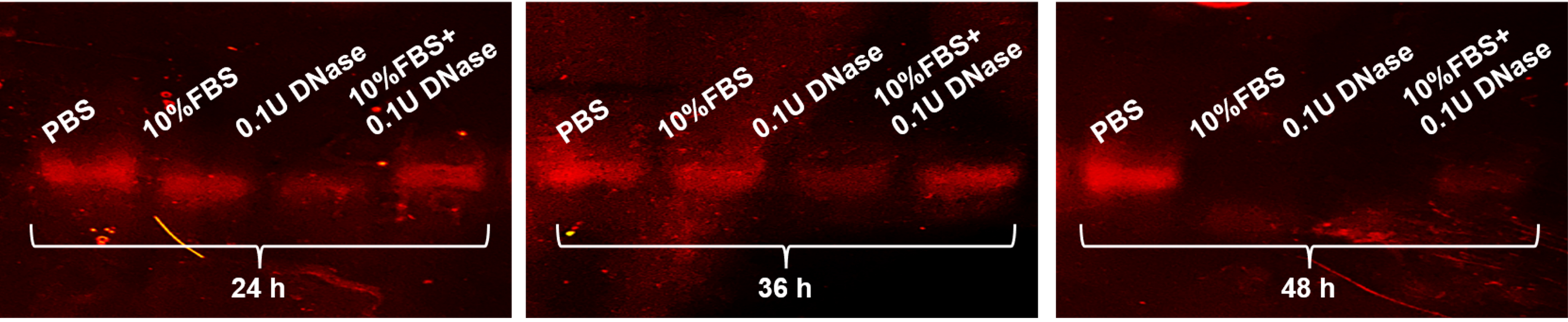

Supplement: Supplementary 1 — Tables S1 and S2 Figs. S1 to S7 [file research.0295.f1.zip › Figure S2.pdf]

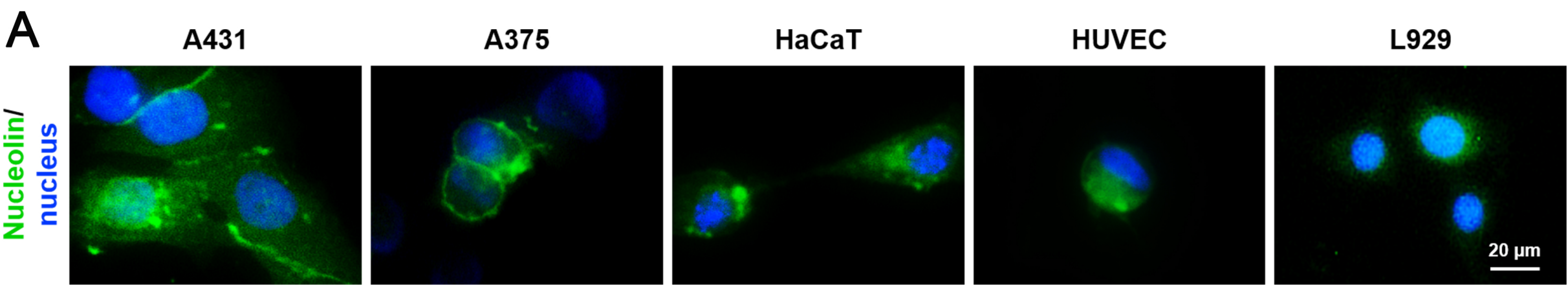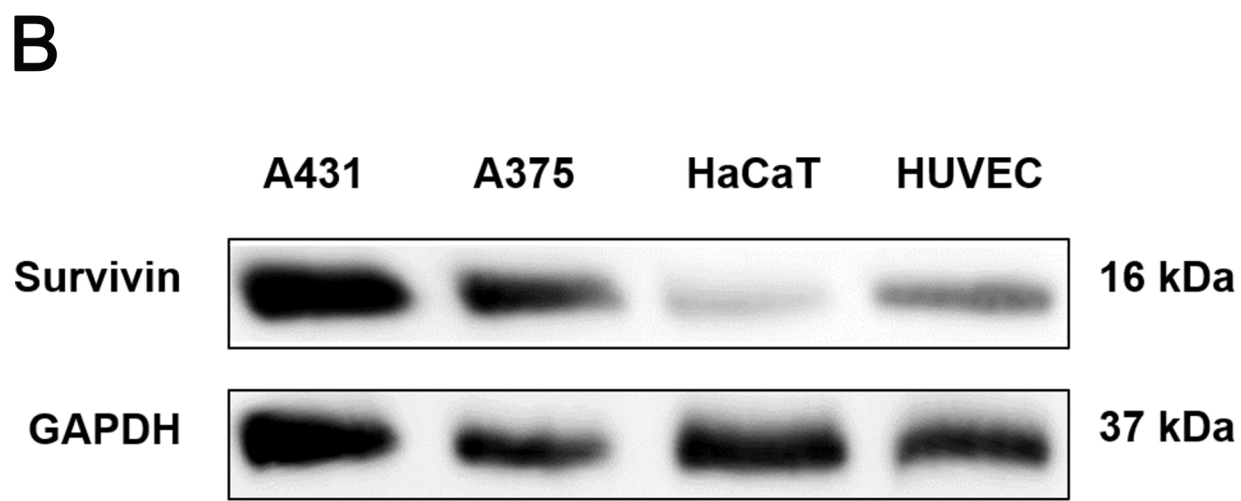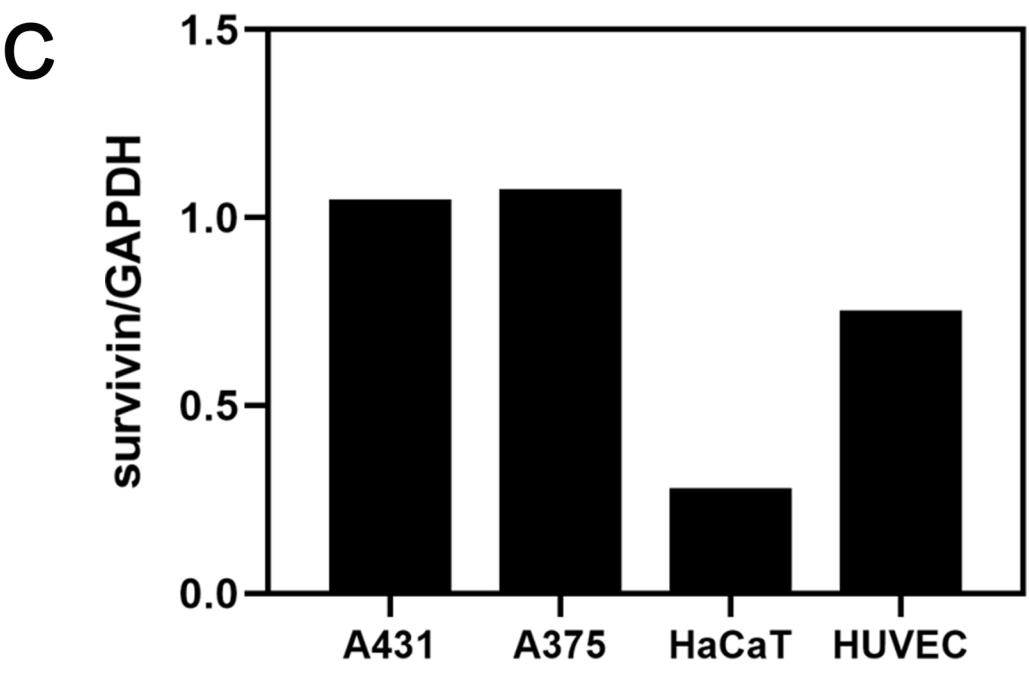

**D**

| proteins             | Cell lines | A431 | A375 | HaCaT | HUVEC | L929 |
|----------------------|------------|------|------|-------|-------|------|
|                      |            |      |      |       |       |      |
| Nucleolin (membrane) |            | ++   | ++   | +     | +     | -    |
| Survivin (human)     |            | +++  | +++  | +     | ++    | -    |

Supplement: Supplementary 1 — Tables S1 and S2 Figs. S1 to S7 [file research.0295.f1.zip › Figure S3.pdf]

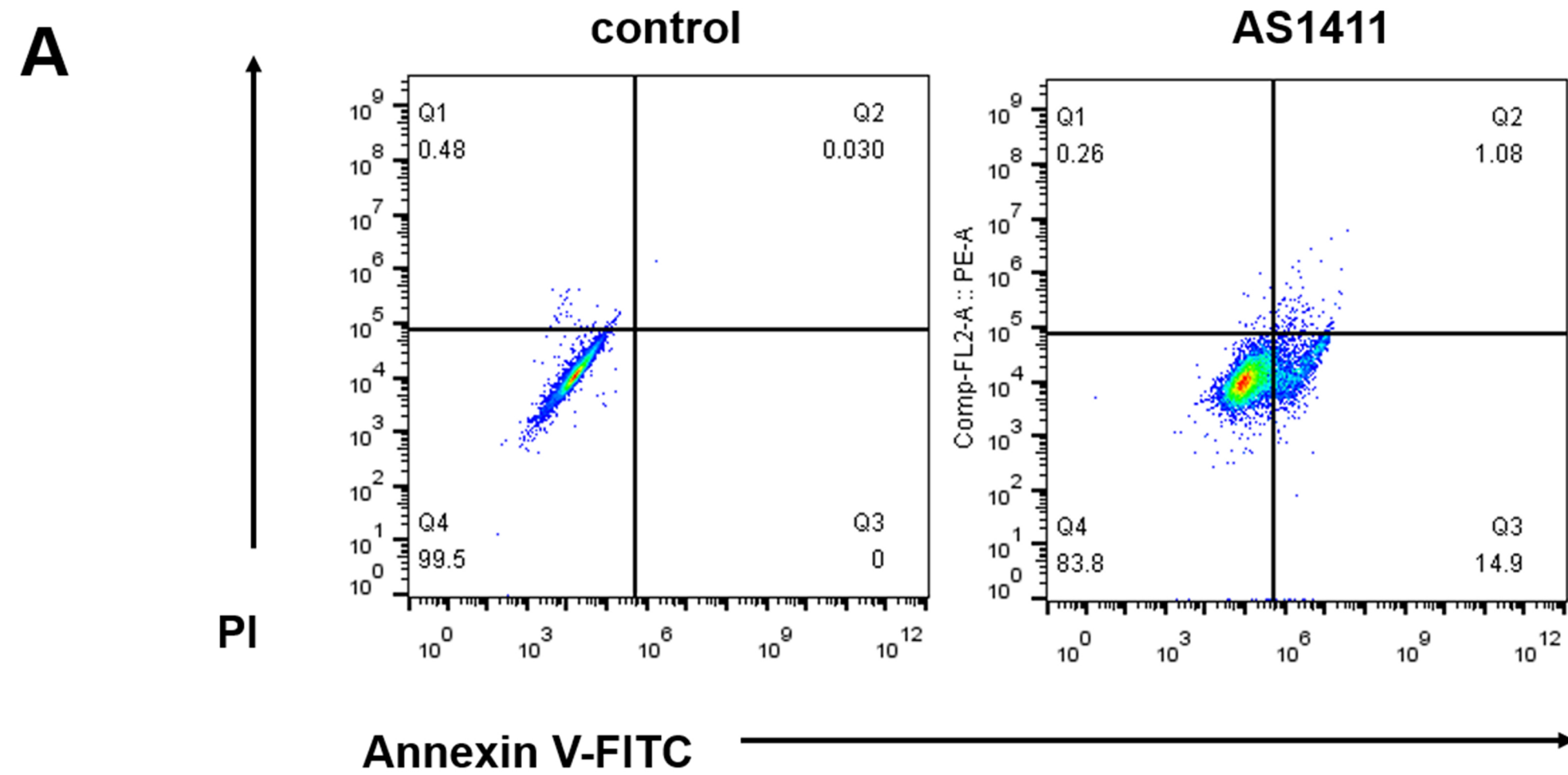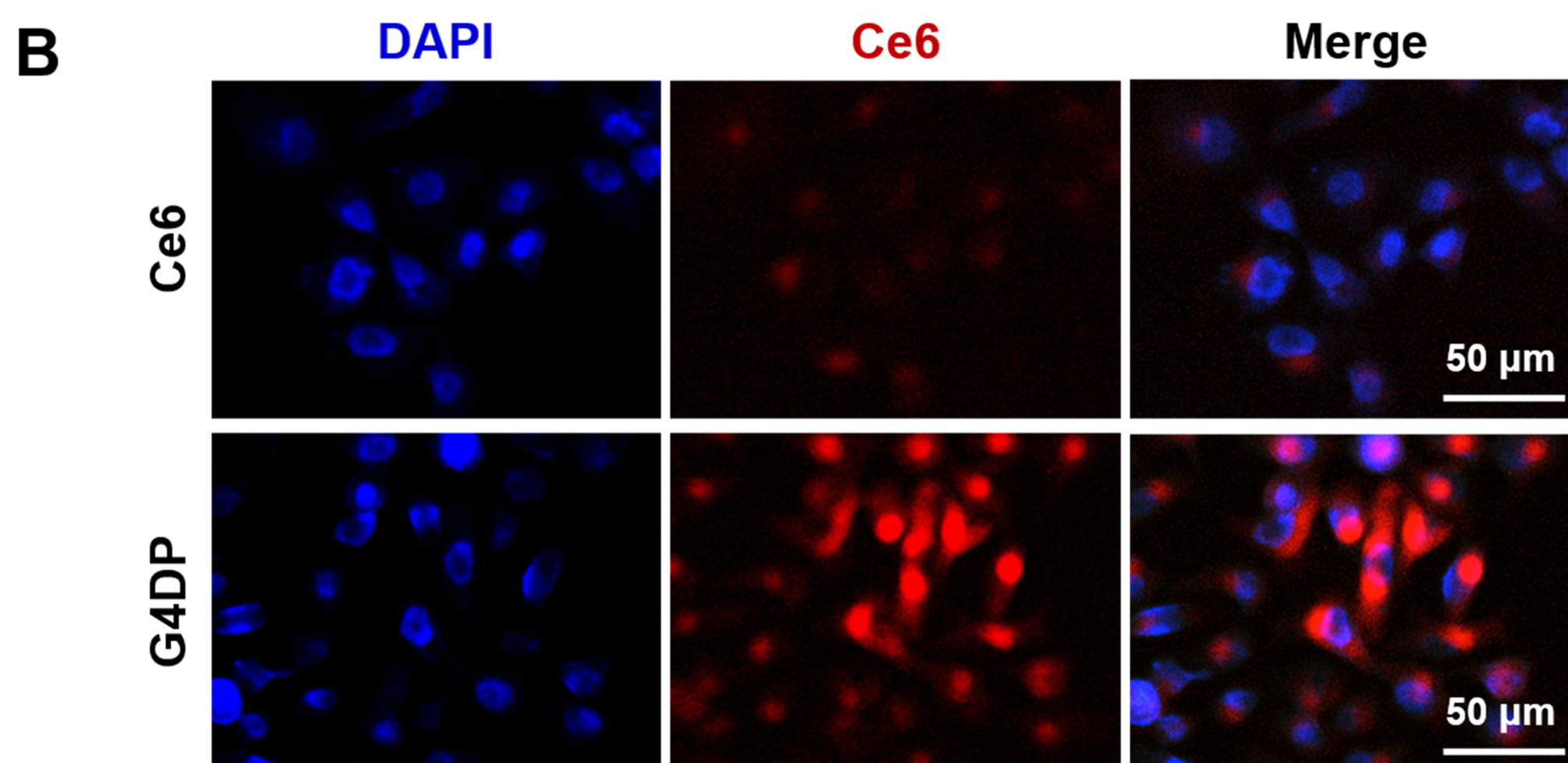

Supplement: Supplementary 1 — Tables S1 and S2 Figs. S1 to S7 [file research.0295.f1.zip › Figure S4.pdf]

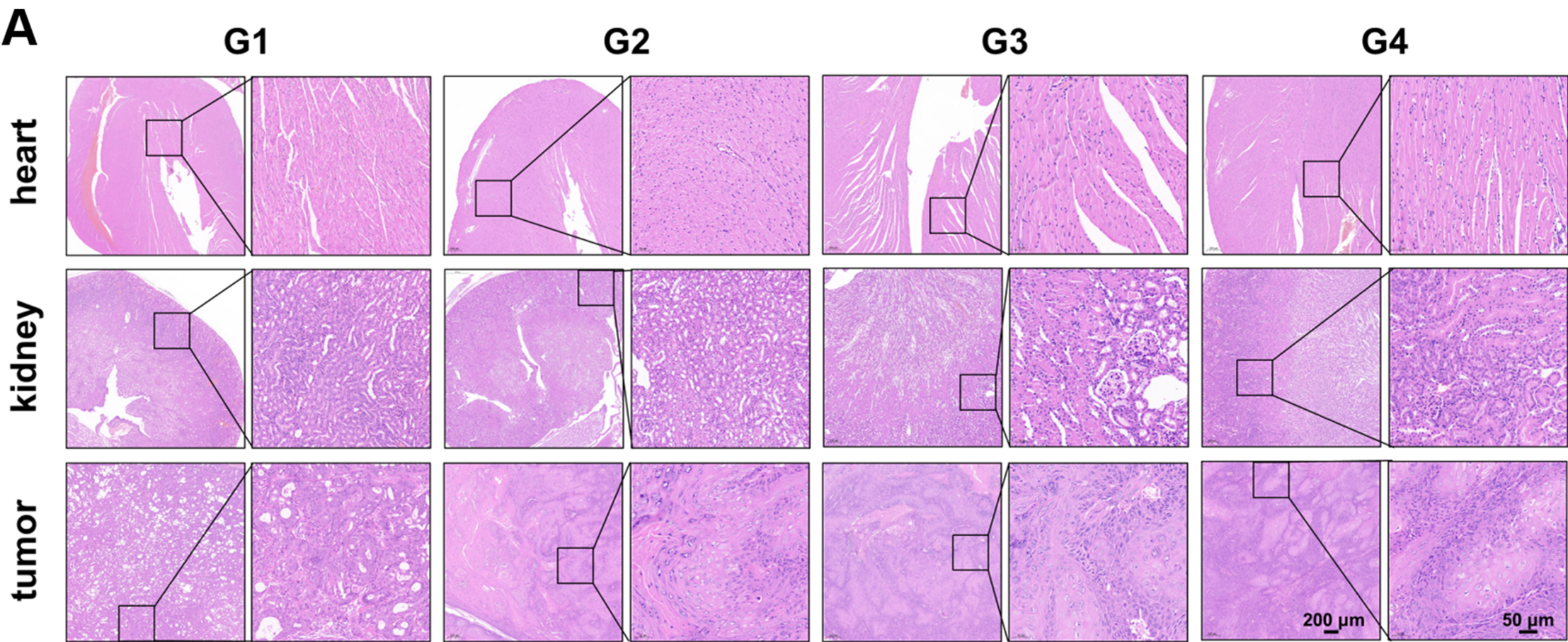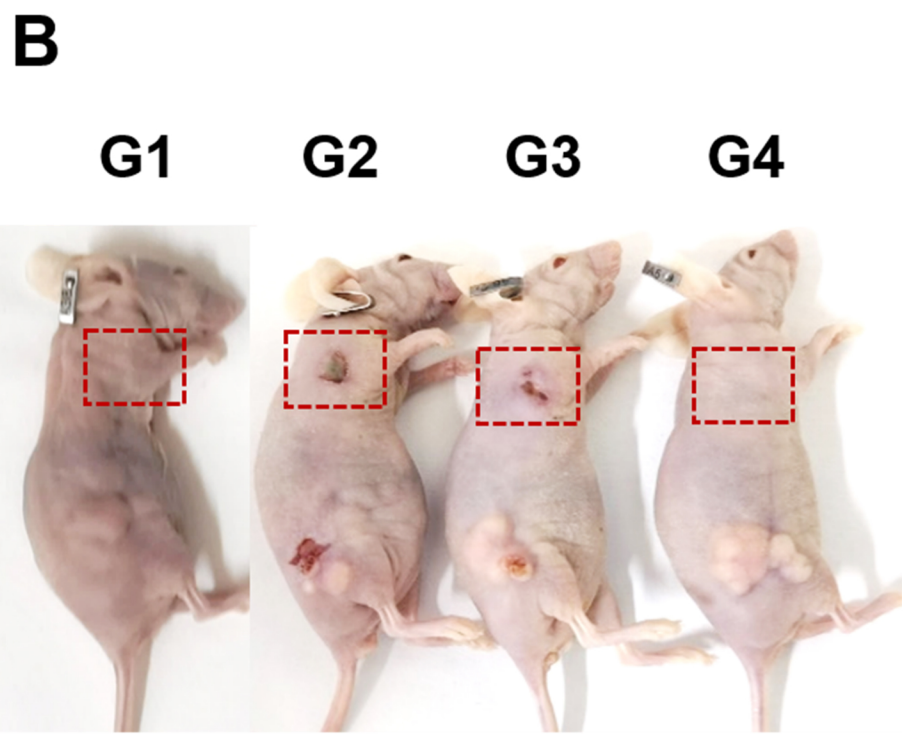

Supplement: Supplementary 1 — Tables S1 and S2 Figs. S1 to S7 [file research.0295.f1.zip › Figure S5.pdf]

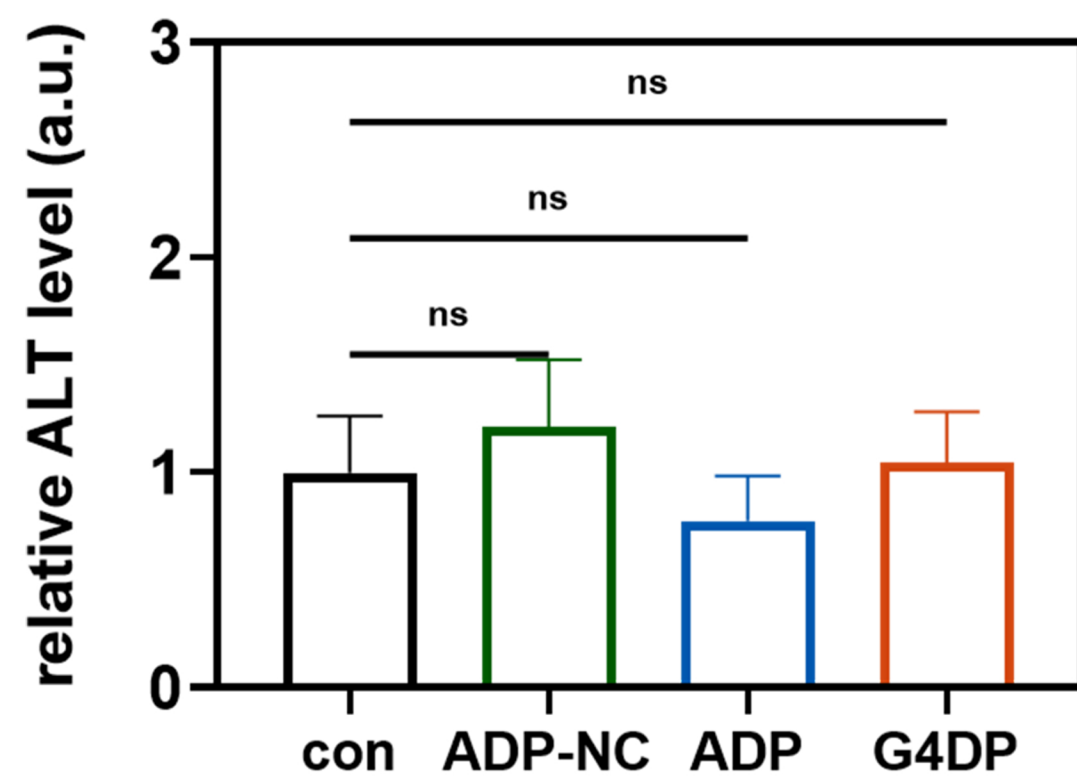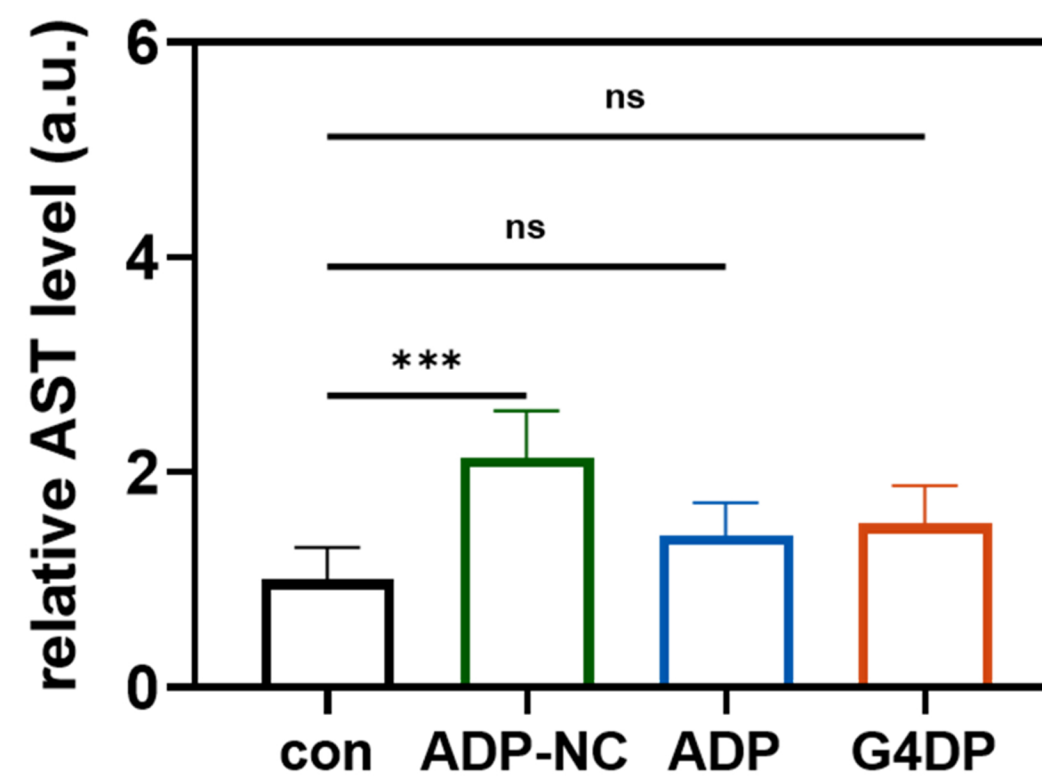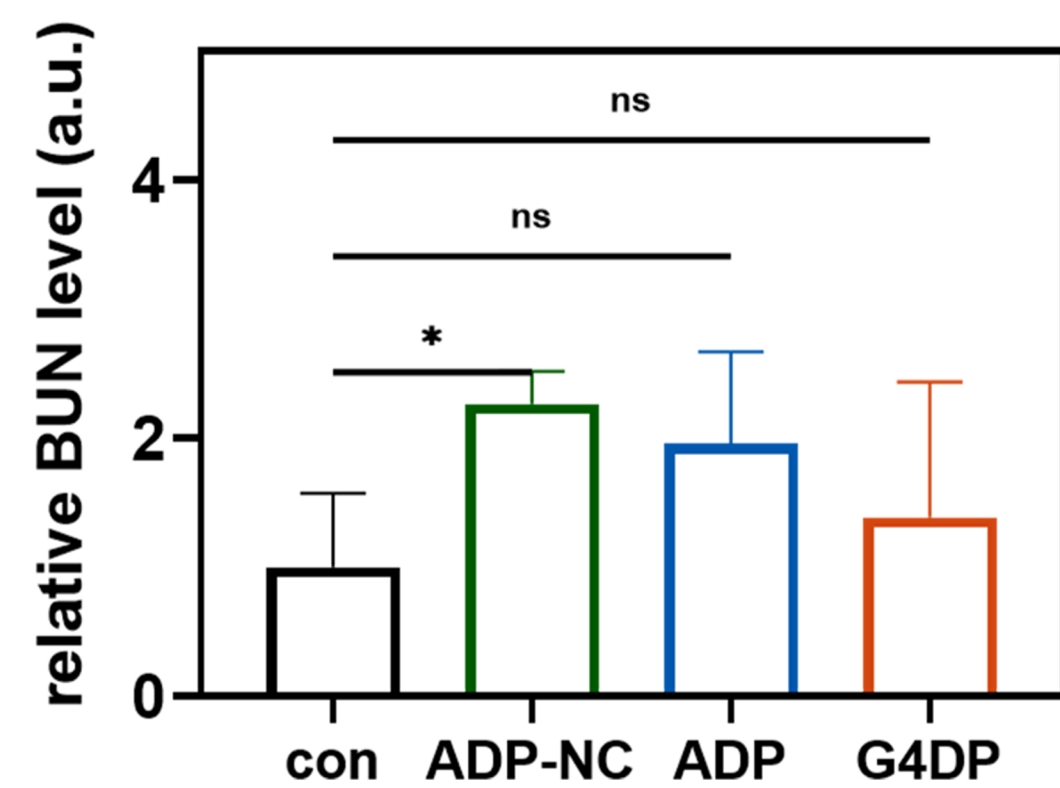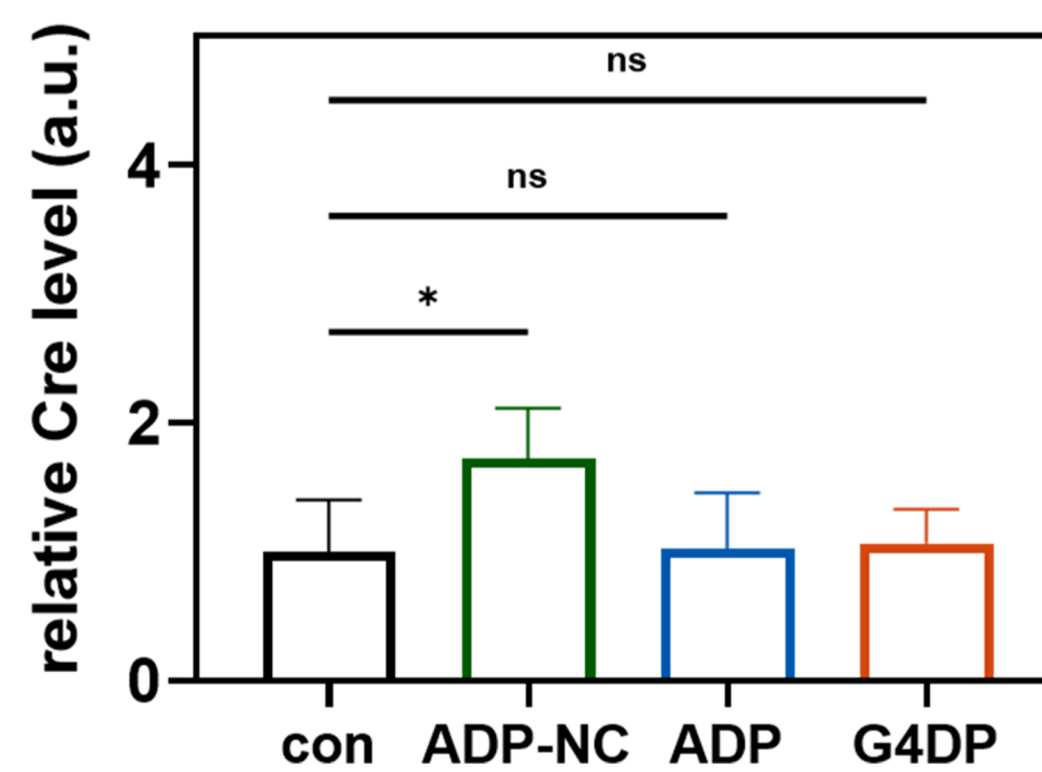

Supplement: Supplementary 1 — Tables S1 and S2 Figs. S1 to S7 [file research.0295.f1.zip › Figure S6.pdf]

**Patient-derived xenografts (PDXs) as model systems for human cancer**

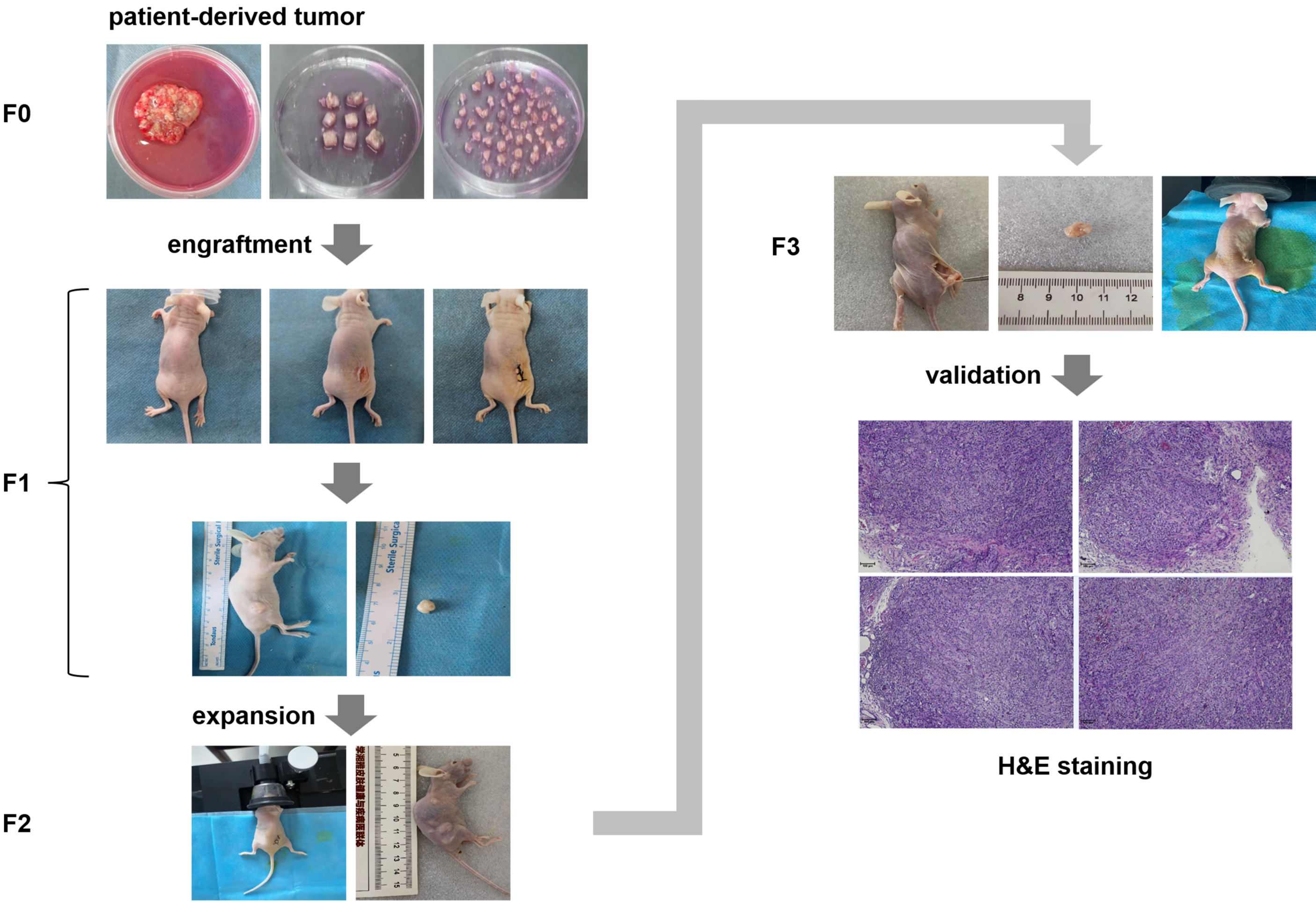

Supplement: Supplementary 1 — Tables S1 and S2 Figs. S1 to S7 [file research.0295.f1.zip › Figure S7.pdf]

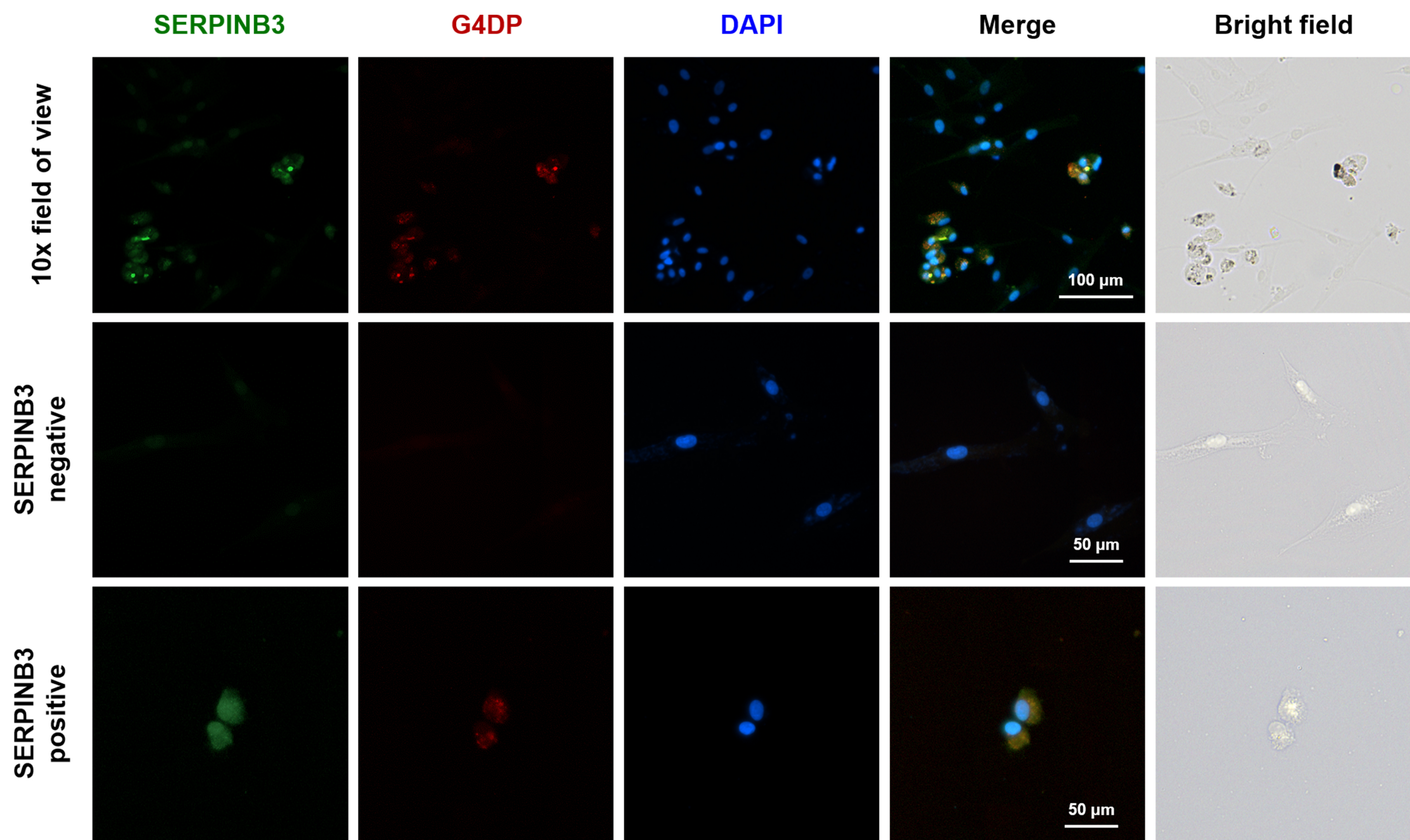

Supplement: Supplementary 1 — Tables S1 and S2 Figs. S1 to S7 [file research.0295.f1.zip › Figure S8.pdf]

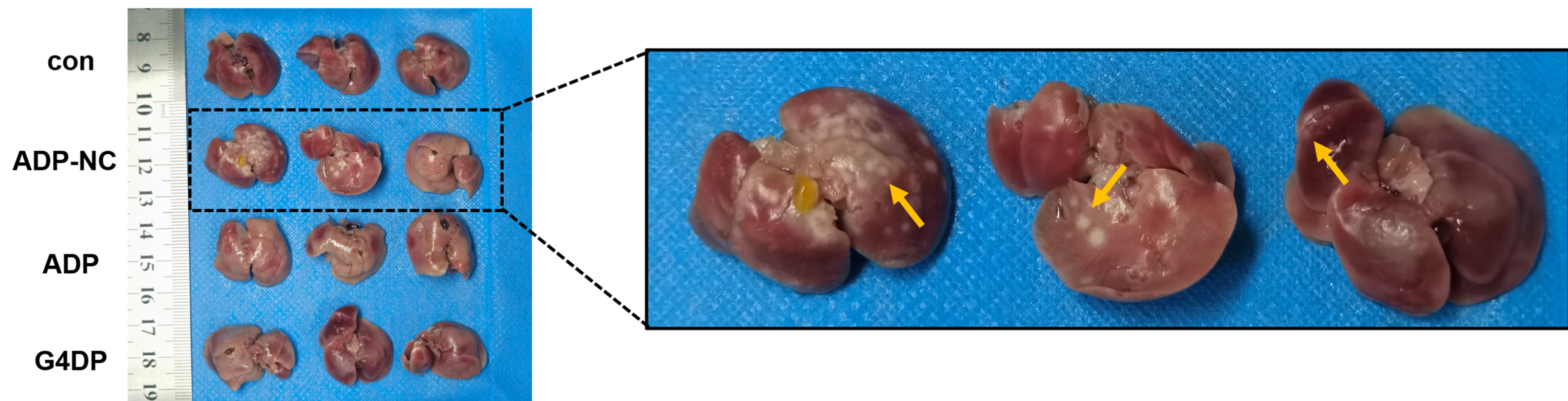

Supplement: Supplementary 1 — Tables S1 and S2 Figs. S1 to S7 [file research.0295.f1.zip › Figure S9.pdf]
